# Expression of PSMA in tumor neovasculature of high grade sarcomas including synovial sarcoma, rhabdomyosarcoma, undifferentiated sarcoma and MPNST

**DOI:** 10.18632/oncotarget.13994

**Published:** 2016-12-16

**Authors:** Birthe Heitkötter, Marcel Trautmann, Inga Grünewald, Martin Bögemann, Kambiz Rahbar, Heidrun Gevensleben, Eva Wardelmann, Wolfgang Hartmann, Konrad Steinestel, Sebastian Huss

**Affiliations:** ^1^ Gerhard Domagk Institute of Pathology, University Hospital Münster, University of Münster, Germany; ^2^ Department of Urology, University Hospital Münster, University of Münster, Germany; ^3^ Department of Nuclear Medicine, University Hospital Münster, University of Münster, Germany; ^4^ Institute of Pathology, University Hospital Bonn, University of Bonn, Germany

**Keywords:** PSMA, sarcoma, neovasculature, soft tissue tumor, therapy

## Abstract

**Aims:**

PSMA (prostate specific membrane antigen) is physiologically expressed in normal prostate tissue. It is overexpressed in prostate cancer cells and has been suggested as a target for antibody-based radioligand therapy. As PSMA expression so far has not been systematically analyzed in soft tissue tumors, the current study aims at investigating a large cohort of different subtypes.

**Methods and Results:**

Immunohistochemistry was used to detect PSMA expression in 779 samples of soft tissue tumors and Ewing sarcoma as a primary bone malignancy. CD34 coexpression was employed to study PSMA expression in the neovasculature. PSMA expression was found in the tumor-associated neovasculature of 151/779 soft tissue/bone tumors (19.38%) and was more frequent in malignant tumors compared to tumors with intermediate or benign biological potential (*p*=0.078). Strong neovascular PSMA expression was predominantly observed in subsets of different sarcomas including 3/20 rhabdomyosarcomas (15%), 4/21 malignant peripheral nerve sheath tumors (19.05%), 6/16 synovial sarcomas (35.29%) and 6/33 undifferentiated pleomorphic sarcomas (18.18%).

**Conclusion:**

We conclude that PSMA is expressed in the neovasculature of a subset of soft tissue tumors to a variable extent. Our observation of strong PSMA expression predominantly occurring in sarcomas might provide a rationale to evaluate PSMA-targeted radioligand therapy in these entities.

## INTRODUCTION

PSMA (prostate specific membrane antigen) is a 100kDa type II transmembrane glycoprotein carrying intra- and extracellular protein domains. Functionally, it exerts a folate hydrolase and neurocarboxypeptidase activity [1, 2]. It was originally found to be physiologically expressed by prostate cells and other tissues (e.g. small intestine, renal tubules or salivary glands) [3, 4]. The discovery of its strong upregulation in prostate cancer cells, however, yielded PSMA-based imaging for the detection of metastatic disease in advanced prostate cancer. PSMA expression was further shown to correlate with prostate cancer grade and was substantiated as an independent predictive factor for tumor recurrence [5]. Moreover, PSMA-based radioligand therapy has been established as a therapeutic regimen in metastasized prostate cancer [2, 6–12].

Surprisingly, PMSA was found to be expressed in the endothelium of tumor-associated neovasculature in some solid malignancies, possibly due to the effect of tumor-associated angiogenic factors [13–17]. Among others, PSMA expression in endothelial neovasculature was shown for clear cell renal carcinomas, transitional cell carcinomas of the urinary bladder, colonic adenocarcinomas, glioblastoma multiforme, lung cancers, malignant melanomas, schwannomas and osteosarcomas [13–15, 18, 19]. In osteosarcomas, PSMA expression in tumor-associated neovasculature furthermore correlated with tumor size, pulmonary metastasis and unfavorable clinical course [19]. The role of PSMA in tumor angiogenesis is part of an autoregulatory loop involving β1-integrin and p21-activated kinase 1 (PAK1). In this loop, active PSMA facilitates endothelial cell invasion through the extracellular matrix by interacting with the cytoskeleton *via* integrin signaling and actin-binding protein Filamin A [8, 20]. These findings have been supported by Gordon *et al*. who reported that PSMA expression is also increased in nonneoplastic, regenerative, and reparative neovasculature (scars, granulation tissue and proliferative endometrium). The authors proposed that the folate hydrolase activity of PSMA enhances the local availability of folic acid, facilitating angiogenesis by increasing the levels of proangiogenic nitric oxide through regeneration of endothelial nitric oxide synthase [8].

Since PSMA expression is not exclusively limited to prostate cancer but also found in the neovasculature of solid epithelial cancers, these results might imply the potential option of new radioligand-based or antiangiogenic therapeutics. As soft tissue tumors have not been systematically analyzed for PSMA expression, the current study aims at investigating a large cohort of different entities, including aggressive subgroups of tumors.

## RESULTS

### PSMA expression in tumor neovasculature

PSMA expression in the tumor-associated neovasculature was found to be positive in 151 of 779 soft tissue/bone tumors (19.38%): 2/67 well differentiated liposarcomas (2.99%), 17/75 dedifferentiated liposarcomas (22.67%), 5/10 pleomorphic liposarcomas (50%), 1/30 myxoid liposarcomas (3.34%), 0/2 lipomas (0%), 1/7 embryonal rhabdomyosarcomas (14.29%), 3/5 pleomorphic rhabdomyosarcomas (60%), 1/8 alveolar rhabdomyosarcomas (12.5%), 0/6 leiomyomas (0%), 21/66 leiomyosarcomas (31.81%), 2/6 haemangiomas (33.34%), 3/29 angiosarcomas of soft tissue (10.34%), 3/14 schwannomas (21.43%), 7/21 malignant peripheral nerve sheath tumors (33.34%), 0/2 neurofibromas (0%), 0/2 ganglioneuromas (0%), 1/6 myxofibrosarcomas (16.67%), 12/35 extrapleural solitary fibrous tumors (34.29%), 0/2 inflammatory myofibroblastic tumors (0%), 10/44 desmoid-type fibromatosis (22.73%), 14/106 Ewing sarcomas (13.21%), 23/183 gastrointestinal stromal tumors (12.57%), 9/16 synovial sarcomas (56.25%), 15/33 undifferentiated pleomorphic sarcomas and 1/4 endometrial stromal sarcomas (25%). Of these tumors, the majority (108/779; 13.86%) showed low expression levels while 5.52% percent (43/779) showed strong PSMA expression (see Table 2 and Figure 2). Detailed data on staining intensity and distribution patterns for each evaluated tumor is shown in Supplementary Table 1.

**Table 1:** TMA composition with the numbers of cases included (n=779)

Subgroup	Entity
Adipocytic	Lipoma (2) Liposarcoma (152) • Well differentiated 67) • Dedifferentiated (75) • Pleomorphic (10) Myxoid liposarcoma (30)
Skeletal-muscle tumor*	Rhabdomyosarcoma (20) • embryonal (7) • alveolar (8) • pleomorphic (5)
Smooth-muscle tumor	Leiomyoma (6) Leiomyosarcoma (66)
Vascular tumor	Haemangioma (6) Angiosarcoma of soft tissue (29)
Nerve sheath tumor	Schwannoma (14) Neurofibroma (2) Ganglioneuroma (2) Malignant peripheral nerve sheath tumor (21)
Fibroblastic/myofibroblastic tumors	Inflammatory myofibroblastic tumor (2) Extrapleural solitary fibrous tumor (35) Desmoid-type fibromatosis (44) Myxofibrosarcoma (6)
Gastrointestinal stromal tumor	Gastrointestinal stromal tumor (183)
Tumor of uncertain differentiation	Synovial Sarcoma (16)
Undifferentiated sarcomas	Undifferentiated pleomorphic sarcoma (33) Endometrial stromal sarcoma (4)
Primary bone tumors	Ewing-Sarcoma (106)

**Table 2:** Numbers of tumors with strong PSMA expression in the neovasculature (PSMA labelling index = 2; total n = 43)

Subgroup	n	Entity
Adipocytic	2	0/2 Lipoma 0/67 Well differentiated liposarcoma 0/75 Dedifferentiated liposarcoma **2/20 Pleomorphic liposarcoma** 0/30 Myxoid liposarcoma
Skeletal-muscle	3	0/7 Embryonal rhabdomyosarcoma 1/8 Alveolar rhabdomyosarcoma* 2/5 Pleomorphic rhabdomyosarcoma
Smooth-muscle	7	0/6 Leiomyoma **7/66 Leiomyosarcoma**
Vascular tumor	3	0/6 Haemangioma **3/29 Angiosarcoma of soft tissue**
Nerve sheath tumor	5	1/14 Schwannoma 0/2 Neurofibroma 0/2 Ganglioneuroma **4/21 Malignant peripheral nerve sheath tumor**
Fibroblastic/myofibroblastic tumors	3	0/2 Inflammatory myofibroblastic tumor 1/35 Extrapleural solitary fibrous tumor 2/44 Desmoid-type fibromatosis 0/6 Myxofibrosarcoma
Gastrointestinal stromal tumor	2	2/183 Gastrointestinal stromal tumor
Tumor of uncertain differentiation	6	6/16 Synovial sarcoma
Undifferentiated sarcomas	6	6/33 Undifferentiated pleomorphic sarcoma 0/4 Endometrial stromal sarcoma
Primary bone tumors	6	6/106 Ewing-Sarcoma

Entities with more than one tumor and strong PSMA expression in at least 10% of the tumor’s neovasculature are indicated in bold.

* In one rhabdomyosarcoma a strong tumor cell expression was observed.

This entity was therefore analyzed in a validation study containing further 16 rhabdomyosarcomas.

No additional case showing a tumor cell expression was found.

**Figure 1:**
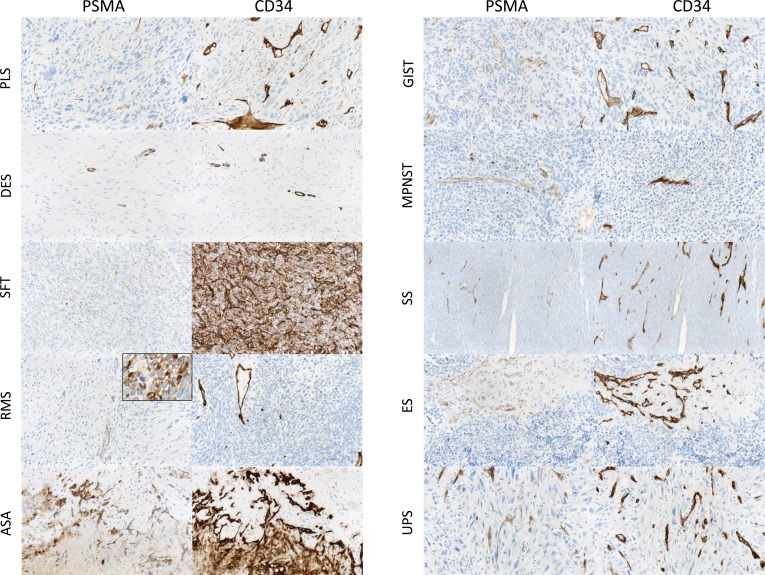
PSMA expression in the neovasculature of sarcomas Neovascular PSMA expression in different sarcoma subtypes. Vasculature was identified by means of CD34 coexpression. (PLS, pleomorphic liposarcoma; DES, desmoid-type fibromatosis; SFT, solitary fibrous tumor; RMS, rhabdomyosarcoma; ASA, angiosarcoma; GIST, gastrointestinal stromal tumor; MPNST, malignant peripheral nerve sheath tumor; SS, synovial sarcoma; ES, Ewing sarcoma; UPS, undifferentiated pleomorphic sarcoma). In one case of RMS a significant PSMA expression of tumor cells (inset) was observed.

**Figure 2:**
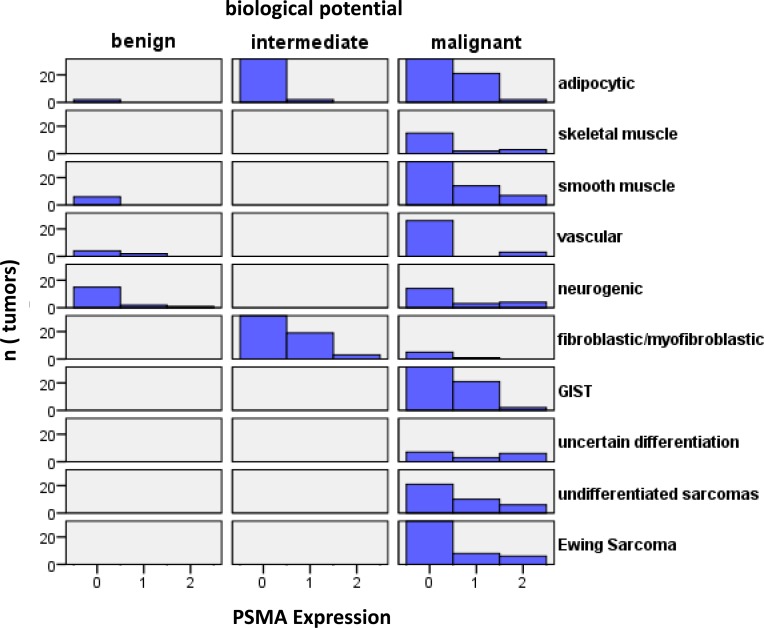
Histograms of tumors according to their biological potential and PSMA labelling index Histograms of tumors according to their biological potential and PSMA labeling index.

We then performed a histogram analysis linking the biological potential of different tumors with the neovascular PSMA labelling index (Figure 2). The overall PSMA expression (low and high expression, labelling index 1 and 2) was found in the neovasculature of 122/599 (20.37%) malignant tumors, whereas PSMA expression in the neovasculature of tumors with intermediate biological potential (24/148 (16.22%) and benign tumors (5/32 (15.63%)) tended to be lower (*p* = 0.237, *Fisher´s exact test*, malignant *vs*. intermediate/benign tumors).

Analyzing only tumors with strong neovascular PSMA expression (labelling index 2), malignant tumors with a high PSMA expression were more frequent (39/599; 6.51%) compared to tumors of intermediate biological potential (3/148; 2.03%) and benign tumors (1/32; 3.13%) (*p* = 0.078, *Fisher´s exact test*, malignant *vs*. intermediate/benign tumors).

According to the FNCLCC grading of sarcomas, some tumors are designated as high grade sarcomas (grade 3) by diagnosis, e.g. Ewing sarcomas, rhabdomyosarcomas (except spindle cell and botryoid variants), angiosarcomas and pleomorphic liposarcomas. Focusing only on the PSMA expression in the neovasculature of these high grade sarcomas, we noted that the neovasculature of 2/20 pleomorphic liposarcomas (10%), 3/29 angiosarcomas of the soft tissue (10.34%), 1/8 alveolar rhabdomyosarcomas (12.50%) and 2/5 pleomorphic rhabdomyosarcomas (40%) showed a strong expression (labelling index = 2) in more than 10% of the investigated cases, whereas only 6/106 (5.66%) of the investigated Ewing sarcomas presented a strong expression (labelling index = 2) in the tumor-associated neovasculature.

Regarding all represented entities, the highest percentage of a strong PSMA expression (labelling index = 2) could be found in the neovasculature of pleomorphic rhabdomyosarcomas (40%), followed by synovial sarcomas (37.50%), malignant peripheral nerve sheath tumors (19.05%), undifferentiated pleomorphic sarcomas (18.19%), alveolar rhabdomyosarcomas (12.50%), leiomyosarcomas (10.61%), angiosarcomas of the soft tissue (10.34%) and pleomorphic liposarcomas (10%).

### PSMA expression in tumor cells

We observed a very strong cellular PSMA expression in one case of alveolar rhabdomyosarcoma (inset Figure 1). This alveolar rhabdomyosarcoma was found in the right upper lung lobe of a 83 year old male patient. Before undergoing surgery, the lesion was thought to be non-small cell lung cancer. The patient had no history of prostate cancer and his PSA-levels were normal. Immunohistological staining showed no expression of cytokeratins (AE1/3, CK7, CK18) or S-100 and a weak expression of CD56. There was a strong expression of vimentin and desmin as well as a nuclear positivity for Myo-D1 and Myogenin. Staining against caldesmon and smooth muscle actin was negative. A translocation involving the *FOXO1* gene on chromosome 13q14 could be excluded by fluorescence *in situ* hybridization (FISH). The tumor was finally signed out as translocation-negative alveolar rhabdomyosarcoma (solid variant).

### Representativeness of tissue microarrays

To rule out a selection bias by IHC analysis of tissue microarrays (TMAs), we performed additional stainings of whole slides from 12 tumors of benign biological potential (lipoma and hemangioma), 13 tumors with intermediate biological potential (desmoid type fibromatosis) and 12 high grade sarcomas (synovial sarcoma and MPNST) (Supplementary Table 2). PSMA staining of tumor-associated neovasculature could be observed in two additional whole slide cases (5.4%) that had previously been classified as “negative” based on the TMA staining. There were no differences with regard to the rate of PSMA staining of tumor cells.

## DISCUSSION

Apart from its known strong cellular expression in prostate cancer, PSMA is expressed in the tumor neovasculature of different solid epithelial cancer subtypes. This finding prompted us to systematically analyze a large cohort of different soft tissue tumors for neovascular as well as intratumoral PSMA expression. We found strong neovascular PSMA expression in a subset of different malignant soft tissue tumors including pleomorphic liposarcoma, rhabdomyosarcoma, leiomyosarcoma, angiosarcoma, MPNST, synovial sarcoma and undifferentiated sarcoma. In addition, one case of rhabdomyosarcoma showed cytoplasmic PSMA expression. Overall, neovascular PSMA expression was more frequent in malignant tumors than in tumors with intermediate or benign biological potential. However, in schwannomas, we found neovascular PSMA-expression in three of 14 cases (21.42%). A similar finding was reported by Wang *et al*. who found an expression of PSMA in the neovasculature of schwannomas in nine of 11 cases [18].

Today, we distinguish over 50 histological types of soft tissue sarcomas with distinct histological appearance and clinical behavior [21]. Especially in advanced disease, clinical management of soft tissue sarcomas requires a multidisciplinary approach [22]. Representing the most reproducible and reliable prognostic factor for survival, complete (R0) resection remains the cornerstone of treatment for resectable soft tissue sarcomas. Therefore, the only curative loco-regional approach as well as the usual first-line treatment of these tumors is wide margin surgery. Radiotherapy represents a further therapeutic tool shown to significantly improve outcome of patients with soft tumors. In contrast, the therapeutic role of conventional chemotherapy in advanced soft tissue sarcomas is limited. Anthracycline-based chemotherapy, which is the first-line treatment for most advanced soft tissue sarcomas [22], has a response rate of only ~26 % [23]. Response rates for Ifosfamide (with or without doxorubicin), another first-line treatment, are as low as ~25 % [24]. After failure of first-line therapy, gemcitabine/docetaxel offers another therapeutic possibility but treatment options beyond this approach are rare [22]. In metastatic disease, a conventional-dose, single-agent chemotherapy is executed [22]. Due to the lack of reproducible impact on survival (in part because trials include only small numbers of patients with heterogeneous groups of histological/molecular subtypes) the effectiveness of adjuvant chemotherapy after resection of high-grade soft tissue sarcomas remains controversial. In a few trials, a lower risk for local recurrence was observed among patients receiving adjuvant chemotherapy, however, without any significant gain in overall survival [25].

In the light of these findings, there is an urgent need for new therapeutic options, especially for advanced soft tissue sarcomas. Since PSMA is expressed in the neovasculature of different solid epithelial cancers and represents a possible target for radioligand-based or antiangiogenic therapeutic strategies, we aimed at investigating PSMA expression in soft tissue and bone tumors as well as in the tumor-associated neovasculature. Our idea was supported by two recent studies evaluating PSMA-targeted therapies in different solid cancer subtypes.

In a phase I trial Milowsky *et al*. demonstrated that tumor-associated neovasculature in multiple advanced metastatic solid cancers could be targeted with an Indium-111- labeled antibody (J591) binding the extracellular domain of PSMA. Imaging with a chest x-ray, computed tomography scan or magnetic resonance imaging (MRI) by tumor type was as follows: 7/10 kidney (70%), 4/4 colorectal (100%), 3/3 lung (100%), 1/1 bladder (100%), 3/3 pancreas (100%) and 1/1 melanoma (100%). However, no objective tumor regression could be observed whereas toxicity was acceptable [26].

Another phase I study using the PSMA-targeted docetaxel-containing nanoparticle BIND-014 in patients with advanced solid tumors was recently performed [27]. BIND-014 showed noteworthy activity in multiple tumor types leading to complete response in one case, a 46-year-old female with cervical cancer metastatic to lymph nodes. Radiographically confirmed partial responses could be detected in an ampullary adenocarcinoma metastatic to the liver, in a *KRAS*-mutant lung adenocarcinoma and in cases of breast and gastroesophageal cancer. The authors also reported that several BIND-014-sensitive tumors displayed a moderate to high expression of PSMA, while others did not. The authors concluded that high PSMA expression increases uptake of BIND-014 in certain tumors, pointing towards a potential utility of PSMA expression as a predictive biomarker for responsiveness to BIND-014.

For patients with prostate cancer, the PSMA-targeted radionuclide therapy has been shown to be a therapeutic and diagnostic option [6, 28]. PSMA-617 seems to be the most promising ligand for diagnostics and therapy of prostate cancer metastases and recurrences. Developed by the German Cancer Research Centre (DKFZ) in Heidelberg, PSMA-617 is an important theranostic compound [29]. Several studies using Lutetium-177 labeled PSMA-617 in patients with metastatic castrate resistant prostate cancer have shown respectable response values and acceptable toxicity profile [7, 28, 30]. Future studies using Lutetium-177-PSMA-617 complementary to established therapeutics or randomized placebo controlled trials have to evaluate the therapeutic effect of this new agent in response and survival of patients with metastatic prostate cancer.

In the light of these different studies, our immunohistochemical finding of strong neovascular PSMA expression in a subset of different malignant soft tissue sarcomas seems promising, since it points towards a possible use of PSMA-targeted radioligand or antibody-based antiangiogenic therapy for these aggressive tumors. The actual rate of sarcomas with PSMA-expressing neovasculature might even be slightly higher, since we found a low rate of false-negative cases when immunohistochemistry is performed on TMAs instead of whole slides. The results of our study support first in human imaging studies to evaluate tracer uptake and retention in the most promising sarcoma entities. In the event of high PSMA uptake and long retention in tumor tissue this might then point towards further studies for the evaluation of radioligand therapy using radiolabeled PSMA ligands.

## MATERIALS AND METHODS

### Tissue microarrays (TMAs)

We used 23 different TMAs containing at least two representative cores (core diameter 1 mm) of different soft tissue tumors. Selected tumor areas were confirmed by two experienced pathologists before and after TMA construction. As one rhabdomyosarcoma case displayed a strong PSMA tumor cell expression, we decided to select 16 additional rhabdomyosarcomas cases from our archival files. For these cases, whole slides were used to study the expression of PSMA. In total, 779 different tumors were investigated (Table 1). To address the question whether the rate of PSMA positive samples is systematically underestimated by the TMA approach we analyzed a small subset of tumors (*n* = 37) both on TMA and whole slide sections (Supplementary Table 2). The study was approved by the local ethics committee (Az. 2016-091-f-S).

### Immunohistochemistry

Immunhistochemistry (IHC) was performed on 4-μm-thick paraffin sections using the peroxidase-conjugated avidin-biotin method. Antibodies included a monoclonal mouse anti-PSMA antibody (clone 3E6, Ventana, Germany, 1:50 dilution) and a monoclonal anti-CD34 antibody (clone QBEnd10, Ventana, Germany, ready to use concentration of 0.8μg/ml). In brief, sections were deparaffinized in xylene and rehydrated through graded ethanol at room temperature. Incubation with the primary antibodies was performed for 30 minutes at room temperature. After washing, the sections were incubated with biotinylated secondary antibodies. Immunoreactions were visualized using a 3-amino-9-ethylcarbazole as a substrate (Ventana Optiview DAB IHC detection KIT, Ref: 760-700, Germany). Prostate carcinoma tissue sections served as a positive control. Specificity of the PSMA antibody was demonstrated by western immunoblotting of 22RV1 prostate cancer whole cell lysate (data not shown).

### Assessment of PSMA expression

PSMA expression was evaluated by two experienced pathologists (BH and SH) on immunostained TMA slides. Tumor cells and associated neovascular endothelium were analyzed separately and the identity of vascular structures was confirmed by CD34 coexpression, a common marker for endothelial cells [31–33].

Any reactivity, either in tumor cells or neoplastic vessels, was considered positive. Staining intensity was scored semiquantitatively as negative (0), weak (1 = barely perceptible staining at high power (400x) magnification), moderate (2 = readily apparent at low power (40x) magnification) or strong (3). The fraction of PSMA positive cells was assessed as < 5% or > 5%. In the case of heterogeneous staining, the predominant pattern was recorded. For further analysis, labeling indices were defined. A weak (1) or moderate (2) staining intensity in < 5% of the neovasculature and a weak (1) staining intensity in > 5% of the neovasculature was allocated to the “low expression” group (PSMA labelling index = 1), whereas a moderate (2) staining intensity in > 5% of the neovasculature and a strong (3) staining intensity in < or > 5% of the neovasculature were assigned to the “strong expression” group (PSMA labelling index = 2). This scoring system has been previously established in mesenchymal tumors [34].

### Statistics

SPSS 21 software (IBM, Armonk, NY, USA) was used. Fisher’s exact was used if appropriate. All tests were two-sided with a 95% confidence interval.

## SUPPLEMENTARY TABLES




