# Supplementary material for: Expression of PSMA in tumor neovasculature of high grade sarcomas including synovial sarcoma, rhabdomyosarcoma, undifferentiated sarcoma and MPNST
Source: Oncotarget. 2016 Dec 16;8(3):4268–76. doi: 10.18632/oncotarget.13994 (PMC5354830; doi:10.18632/oncotarget.13994)
Supplement: Supplementary file 1 [file oncotarget-08-4268-s001.docx]

**Supplementary Table S1.** Semi-quantitative immunohistochemistry results

|  |  |  | **PSMA Immunohistochemistry** | | | | | | |
| --- | --- | --- | --- | --- | --- | --- | --- | --- | --- |
| **No.** | **WHO subtype** | **Tumor type** | **Neovasculature proportion*** | **Neovasculature intensity [0-3]** |  | **Tumor cells proportion*** | **Tumor cells intensity [0-3]** |  |  |
| 1 | Adipocytic | WDLS | 0 | 0 |  | 0 | 0 |  |  |
| 2 | Adipocytic | WDLS | 0 | 0 |  | 0 | 0 |  |  |
| 3 | Adipocytic | WDLS | 0 | 0 |  | 0 | 0 |  |  |
| 4 | Adipocytic | WDLS | 0 | 0 |  | 0 | 0 |  |  |
| 5 | Adipocytic | WDLS | 0 | 0 |  | 0 | 0 |  |  |
| 6 | Adipocytic | WDLS | 0 | 0 |  | 0 | 0 |  |  |
| 7 | Adipocytic | WDLS | 0 | 0 |  | 0 | 0 |  |  |
| 8 | Adipocytic | WDLS | 0 | 0 |  | 0 | 0 |  |  |
| 9 | Adipocytic | WDLS | 1 | 1 |  | 0 | 0 |  |  |
| 10 | Adipocytic | WDLS | 0 | 0 |  | 0 | 0 |  |  |
| 11 | Adipocytic | WDLS | 0 | 0 |  | 0 | 0 |  |  |
| 12 | Adipocytic | WDLS | 0 | 0 |  | 0 | 0 |  |  |
| 13 | Adipocytic | WDLS | 0 | 0 |  | 0 | 0 |  |  |
| 14 | Adipocytic | WDLS | 0 | 0 |  | 0 | 0 |  |  |
| 15 | Adipocytic | WDLS | 0 | 0 |  | 0 | 0 |  |  |
| 16 | Adipocytic | WDLS | 0 | 0 |  | 0 | 0 |  |  |
| 17 | Adipocytic | WDLS | 0 | 0 |  | 0 | 0 |  |  |
| 18 | Adipocytic | WDLS | 0 | 0 |  | 0 | 0 |  |  |
| 19 | Adipocytic | WDLS | 0 | 0 |  | 0 | 0 |  |  |
| 20 | Adipocytic | WDLS | 0 | 0 |  | 0 | 0 |  |  |
| 21 | Adipocytic | WDLS | 0 | 0 |  | 0 | 0 |  |  |
| 22 | Adipocytic | WDLS | 0 | 0 |  | 0 | 0 |  |  |
| 23 | Adipocytic | WDLS | 0 | 0 |  | 0 | 0 |  |  |
| 24 | Adipocytic | WDLS | 0 | 0 |  | 0 | 0 |  |  |
| 25 | Adipocytic | WDLS | 0 | 0 |  | 0 | 0 |  |  |
| 26 | Adipocytic | WDLS | 0 | 0 |  | 0 | 0 |  |  |
| 27 | Adipocytic | WDLS | 0 | 0 |  | 0 | 0 |  |  |
| 28 | Adipocytic | WDLS | 1 | 1 |  | 0 | 0 |  |  |
| 29 | Adipocytic | WDLS | 0 | 0 |  | 0 | 0 |  |  |
| 30 | Adipocytic | WDLS | 0 | 0 |  | 0 | 0 |  |  |
| 31 | Adipocytic | WDLS | 0 | 0 |  | 0 | 0 |  |  |
| 32 | Adipocytic | WDLS | 0 | 0 |  | 0 | 0 |  |  |
| 33 | Adipocytic | WDLS | 0 | 0 |  | 0 | 0 |  |  |
| 34 | Adipocytic | WDLS | 0 | 0 |  | 0 | 0 |  |  |
| 35 | Adipocytic | WDLS | 0 | 0 |  | 0 | 0 |  |  |
| 36 | Adipocytic | WDLS | 0 | 0 |  | 0 | 0 |  |  |
| 37 | Adipocytic | WDLS | 0 | 0 |  | 0 | 0 |  |  |
| 38 | Adipocytic | WDLS | 0 | 0 |  | 0 | 0 |  |  |
| 39 | Adipocytic | WDLS | 0 | 0 |  | 0 | 0 |  |  |
| 40 | Adipocytic | WDLS | 0 | 0 |  | 0 | 0 |  |  |
| 41 | Adipocytic | WDLS | 0 | 0 |  | 0 | 0 |  |  |
| 42 | Adipocytic | WDLS | 0 | 0 |  | 0 | 0 |  |  |
| 43 | Adipocytic | WDLS | 0 | 0 |  | 0 | 0 |  |  |
| 44 | Adipocytic | WDLS | 0 | 0 |  | 0 | 0 |  |  |
| 45 | Adipocytic | WDLS | 0 | 0 |  | 0 | 0 |  |  |
| 46 | Adipocytic | WDLS | 0 | 0 |  | 0 | 0 |  |  |
| 47 | Adipocytic | WDLS | 0 | 0 |  | 0 | 0 |  |  |
| 48 | Adipocytic | WDLS | 0 | 0 |  | 0 | 0 |  |  |
| 49 | Adipocytic | WDLS | 0 | 0 |  | 0 | 0 |  |  |
| 50 | Adipocytic | WDLS | 0 | 0 |  | 0 | 0 |  |  |
| 51 | Adipocytic | WDLS | 0 | 0 |  | 0 | 0 |  |  |
| 52 | Adipocytic | WDLS | 0 | 0 |  | 0 | 0 |  |  |
| 53 | Adipocytic | WDLS | 0 | 0 |  | 0 | 0 |  |  |
| 54 | Adipocytic | WDLS | 0 | 0 |  | 0 | 0 |  |  |
| 55 | Adipocytic | WDLS | 0 | 0 |  | 0 | 0 |  |  |
| 56 | Adipocytic | WDLS | 0 | 0 |  | 0 | 0 |  |  |
| 57 | Adipocytic | WDLS | 0 | 0 |  | 0 | 0 |  |  |
| 58 | Adipocytic | WDLS | 0 | 0 |  | 0 | 0 |  |  |
| 59 | Adipocytic | WDLS | 0 | 0 |  | 0 | 0 |  |  |
| 60 | Adipocytic | WDLS | 0 | 0 |  | 0 | 0 |  |  |
| 61 | Adipocytic | WDLS | 0 | 0 |  | 0 | 0 |  |  |
| 62 | Adipocytic | WDLS | 0 | 0 |  | 0 | 0 |  |  |
| 63 | Adipocytic | WDLS | 0 | 0 |  | 0 | 0 |  |  |
| 64 | Adipocytic | WDLS | 0 | 0 |  | 0 | 0 |  |  |
| 65 | Adipocytic | WDLS | 0 | 0 |  | 0 | 0 |  |  |
| 66 | Adipocytic | WDLS | 0 | 0 |  | 0 | 0 |  |  |
| 67 | Adipocytic | WDLS | 0 | 0 |  | 0 | 0 |  |  |
| 68 | Adipocytic | DDLS | 0 | 0 |  | 0 | 0 |  |  |
| 69 | Adipocytic | DDLS | 0 | 0 |  | 0 | 0 |  |  |
| 70 | Adipocytic | DDLS | 0 | 0 |  | 0 | 0 |  |  |
| 71 | Adipocytic | DDLS | 0 | 0 |  | 0 | 0 |  |  |
| 72 | Adipocytic | DDLS | 0 | 0 |  | 0 | 0 |  |  |
| 73 | Adipocytic | DDLS | 0 | 0 |  | 0 | 0 |  |  |
| 74 | Adipocytic | DDLS | 0 | 0 |  | 0 | 0 |  |  |
| 75 | Adipocytic | DDLS | 0 | 0 |  | 0 | 0 |  |  |
| 76 | Adipocytic | DDLS | 1 | 1 |  | 0 | 0 |  |  |
| 77 | Adipocytic | DDLS | 1 | 1 |  | 0 | 0 |  |  |
| 78 | Adipocytic | DDLS | 1 | 2 |  | 0 | 0 |  |  |
| 79 | Adipocytic | DDLS | 0 | 0 |  | 0 | 0 |  |  |
| 80 | Adipocytic | DDLS | 0 | 0 |  | 0 | 0 |  |  |
| 81 | Adipocytic | DDLS | 0 | 0 |  | 0 | 0 |  |  |
| 82 | Adipocytic | DDLS | 0 | 0 |  | 0 | 0 |  |  |
| 83 | Adipocytic | DDLS | 0 | 0 |  | 0 | 0 |  |  |
| 84 | Adipocytic | DDLS | 1 | 1 |  | 0 | 0 |  |  |
| 85 | Adipocytic | DDLS | 1 | 1 |  | 0 | 0 |  |  |
| 86 | Adipocytic | DDLS | 1 | 2 |  | 0 | 0 |  |  |
| 87 | Adipocytic | DDLS | 1 | 2 |  | 0 | 0 |  |  |
| 88 | Adipocytic | DDLS |  |  |  | 0 | 0 |  |  |
| 89 | Adipocytic | DDLS | 0 | 0 |  | 0 | 0 |  |  |
| 90 | Adipocytic | DDLS | 0 | 0 |  | 0 | 0 |  |  |
| 91 | Adipocytic | DDLS | 0 | 0 |  | 0 | 0 |  |  |
| 92 | Adipocytic | DDLS | 0 | 0 |  | 0 | 0 |  |  |
| 93 | Adipocytic | DDLS | 0 | 0 |  | 0 | 0 |  |  |
| 94 | Adipocytic | DDLS | 0 | 0 |  | 0 | 0 |  |  |
| 95 | Adipocytic | DDLS | 1 | 2 |  | 0 | 0 |  |  |
| 96 | Adipocytic | DDLS | 1 | 2 |  | 0 | 0 |  |  |
| 97 | Adipocytic | DDLS | 1 | 1 |  | 0 | 0 |  |  |
| 98 | Adipocytic | DDLS | 1 | 1 |  | 0 | 0 |  |  |
| 99 | Adipocytic | DDLS | 1 | 1 |  | 0 | 0 |  |  |
| 100 | Adipocytic | DDLS | 0 | 0 |  | 0 | 0 |  |  |
| 101 | Adipocytic | DDLS | 0 | 0 |  | 0 | 0 |  |  |
| 102 | Adipocytic | DDLS | 0 | 0 |  | 0 | 0 |  |  |
| 103 | Adipocytic | DDLS | 0 | 0 |  | 0 | 0 |  |  |
| 104 | Adipocytic | DDLS | 0 | 0 |  | 0 | 0 |  |  |
| 105 | Adipocytic | DDLS | 0 | 0 |  | 0 | 0 |  |  |
| 106 | Adipocytic | DDLS | 1 | 1 |  | 0 | 0 |  |  |
| 107 | Adipocytic | DDLS | 1 | 2 |  | 0 | 0 |  |  |
| 108 | Adipocytic | DDLS | 1 | 2 |  | 0 | 0 |  |  |
| 109 | Adipocytic | DDLS | 0 | 0 |  | 0 | 0 |  |  |
| 110 | Adipocytic | DDLS | 0 | 0 |  | 0 | 0 |  |  |
| 111 | Adipocytic | DDLS | 0 | 0 |  | 0 | 0 |  |  |
| 112 | Adipocytic | DDLS | 0 | 0 |  | 0 | 0 |  |  |
| 113 | Adipocytic | DDLS | 0 | 0 |  | 0 | 0 |  |  |
| 114 | Adipocytic | DDLS | 0 | 0 |  | 0 | 0 |  |  |
| 115 | Adipocytic | DDLS | 0 | 0 |  | 0 | 0 |  |  |
| 116 | Adipocytic | DDLS | 0 | 0 |  | 0 | 0 |  |  |
| 117 | Adipocytic | DDLS | 0 | 0 |  | 0 | 0 |  |  |
| 118 | Adipocytic | DDLS | 1 | 2 |  | 0 | 0 |  |  |
| 119 | Adipocytic | DDLS | 1 | 2 |  | 0 | 0 |  |  |
| 120 | Adipocytic | DDLS | 0 | 0 |  | 0 | 0 |  |  |
| 121 | Adipocytic | DDLS | 0 | 0 |  | 0 | 0 |  |  |
| 122 | Adipocytic | DDLS | 0 | 0 |  | 0 | 0 |  |  |
| 123 | Adipocytic | DDLS | 0 | 0 |  | 0 | 0 |  |  |
| 124 | Adipocytic | DDLS | 0 | 0 |  | 0 | 0 |  |  |
| 125 | Adipocytic | DDLS | 0 | 0 |  | 0 | 0 |  |  |
| 126 | Adipocytic | DDLS | 0 | 0 |  | 0 | 0 |  |  |
| 127 | Adipocytic | DDLS | 0 | 0 |  | 0 | 0 |  |  |
| 128 | Adipocytic | DDLS | 0 | 0 |  | 0 | 0 |  |  |
| 129 | Adipocytic | DDLS | 0 | 0 |  | 0 | 0 |  |  |
| 130 | Adipocytic | DDLS | 0 | 0 |  | 0 | 0 |  |  |
| 131 | Adipocytic | DDLS | 0 | 0 |  | 0 | 0 |  |  |
| 132 | Adipocytic | DDLS | 0 | 0 |  | 0 | 0 |  |  |
| 133 | Adipocytic | DDLS | 0 | 0 |  | 0 | 0 |  |  |
| 134 | Adipocytic | DDLS | 0 | 0 |  | 0 | 0 |  |  |
| 135 | Adipocytic | DDLS | 0 | 0 |  | 0 | 0 |  |  |
| 136 | Adipocytic | DDLS | 0 | 0 |  | 0 | 0 |  |  |
| 137 | Adipocytic | DDLS | 0 | 0 |  | 0 | 0 |  |  |
| 138 | Adipocytic | DDLS | 0 | 0 |  | 0 | 0 |  |  |
| 139 | Adipocytic | DDLS | 0 | 0 |  | 0 | 0 |  |  |
| 140 | Adipocytic | DDLS | 0 | 0 |  | 0 | 0 |  |  |
| 141 | Adipocytic | DDLS | 0 | 0 |  | 0 | 0 |  |  |
| 142 | Adipocytic | DDLS | 0 | 0 |  | 0 | 0 |  |  |
| 143 | Adipocytic | PLS | 1 | 1 |  | 0 | 0 |  |  |
| 144 | Adipocytic | PLS | 1 | 1 |  | 0 | 0 |  |  |
| 145 | Adipocytic | PLS | 1 | 2 |  | 0 | 0 |  |  |
| 146 | Adipocytic | PLS | 2 | 3 |  | 0 | 0 |  |  |
| 147 | Adipocytic | PLS | 2 | 3 |  | 0 | 0 |  |  |
| 148 | Adipocytic | PLS | 0 | 0 |  | 0 | 0 |  |  |
| 149 | Adipocytic | PLS | 0 | 0 |  | 0 | 0 |  |  |
| 150 | Adipocytic | PLS | 0 | 0 |  | 0 | 0 |  |  |
| 151 | Adipocytic | PLS | 0 | 0 |  | 0 | 0 |  |  |
| 152 | Adipocytic | PLS | 0 | 0 |  | 0 | 0 |  |  |
| 153 | Adipocytic | MLS | 1 | 1 |  | 0 | 0 |  |  |
| 154 | Adipocytic | MLS | 0 | 0 |  | 0 | 0 |  |  |
| 155 | Adipocytic | MLS | 0 | 0 |  | 0 | 0 |  |  |
| 156 | Adipocytic | MLS | 0 | 0 |  | 0 | 0 |  |  |
| 157 | Adipocytic | MLS | 0 | 0 |  | 0 | 0 |  |  |
| 158 | Adipocytic | MLS | 0 | 0 |  | 0 | 0 |  |  |
| 159 | Adipocytic | MLS | 0 | 0 |  | 0 | 0 |  |  |
| 160 | Adipocytic | MLS | 0 | 0 |  | 0 | 0 |  |  |
| 161 | Adipocytic | MLS | 0 | 0 |  | 0 | 0 |  |  |
| 162 | Adipocytic | MLS | 0 | 0 |  | 0 | 0 |  |  |
| 163 | Adipocytic | MLS | 0 | 0 |  | 0 | 0 |  |  |
| 164 | Adipocytic | MLS | 0 | 0 |  | 0 | 0 |  |  |
| 165 | Adipocytic | MLS | 0 | 0 |  | 0 | 0 |  |  |
| 166 | Adipocytic | MLS | 0 | 0 |  | 0 | 0 |  |  |
| 167 | Adipocytic | MLS | 0 | 0 |  | 0 | 0 |  |  |
| 168 | Adipocytic | MLS | 0 | 0 |  | 0 | 0 |  |  |
| 169 | Adipocytic | MLS | 0 | 0 |  | 0 | 0 |  |  |
| 170 | Adipocytic | MLS | 0 | 0 |  | 0 | 0 |  |  |
| 171 | Adipocytic | MLS | 0 | 0 |  | 0 | 0 |  |  |
| 172 | Adipocytic | MLS | 0 | 0 |  | 0 | 0 |  |  |
| 173 | Adipocytic | MLS | 0 | 0 |  | 0 | 0 |  |  |
| 174 | Adipocytic | MLS | 0 | 0 |  | 0 | 0 |  |  |
| 175 | Adipocytic | MLS | 0 | 0 |  | 0 | 0 |  |  |
| 176 | Adipocytic | MLS | 0 | 0 |  | 0 | 0 |  |  |
| 177 | Adipocytic | MLS | 0 | 0 |  | 0 | 0 |  |  |
| 178 | Adipocytic | MLS | 0 | 0 |  | 0 | 0 |  |  |
| 179 | Adipocytic | MLS | 0 | 0 |  | 0 | 0 |  |  |
| 180 | Adipocytic | MLS | 0 | 0 |  | 0 | 0 |  |  |
| 181 | Adipocytic | MLS | 0 | 0 |  | 0 | 0 |  |  |
| 182 | Adipocytic | MLS | 0 | 0 |  | 0 | 0 |  |  |
| 183 | Adipocytic | Lipoma | 0 | 0 |  | 0 | 0 |  |  |
| 184 | Adipocytic | Lipoma | 0 | 0 |  | 0 | 0 |  |  |
| 185 | SKM | ERMS | 1 | 2 |  | 0 | 0 |  |  |
| 186 | SKM | ERMS | 0 | 0 |  | 0 | 0 |  |  |
| 187 | SKM | ERMS | 0 | 0 |  | 0 | 0 |  |  |
| 188 | SKM | ERMS | 0 | 0 |  | 0 | 0 |  |  |
| 189 | SKM | ERMS | 0 | 0 |  | 0 | 0 |  |  |
| 190 | SKM | ERMS | 0 | 0 |  | 0 | 0 |  |  |
| 191 | SKM | ERMS | 0 | 0 |  | 0 | 0 |  |  |
| 192 | SKM | ARMS | 2 | 3 |  | 70 | 3 |  |  |
| 193 | SKM | ARMS | 0 | 0 |  | 0 | 0 |  |  |
| 194 | SKM | ARMS | 0 | 0 |  | 0 | 0 |  |  |
| 195 | SKM | ARMS | 0 | 0 |  | 0 | 0 |  |  |
| 196 | SKM | ARMS | 0 | 0 |  | 0 | 0 |  |  |
| 197 | SKM | ARMS | 0 | 0 |  | 0 | 0 |  |  |
| 198 | SKM | ARMS | 0 | 0 |  | 0 | 0 |  |  |
| 199 | SKM | ARMS | 0 | 0 |  | 0 | 0 |  |  |
| 200 | SKM | PRMS | 1 | 1 |  | 0 | 0 |  |  |
| 201 | SKM | PRMS | 2 | 2 |  | 0 | 0 |  |  |
| 202 | SKM | PRMS | 1 | 3 |  | 0 | 0 |  |  |
| 203 | SKM | PRMS | 0 | 0 |  | 0 | 0 |  |  |
| 204 | SKM | PRMS | 0 | 0 |  | 0 | 0 |  |  |
| 205 | SMM | LMS | 1 | 1 |  | 0 | 0 |  |  |
| 206 | SMM | LMS | 1 | 1 |  | 0 | 0 |  |  |
| 207 | SMM | LMS | 1 | 1 |  | 0 | 0 |  |  |
| 208 | SMM | LMS | 1 | 1 |  | 2 | 2 |  |  |
| 209 | SMM | LMS | 1 | 1 |  | 0 | 0 |  |  |
| 210 | SMM | LMS | 1 | 1 |  | 0 | 0 |  |  |
| 211 | SMM | LMS | 1 | 1 |  | 0 | 0 |  |  |
| 212 | SMM | LMS | 1 | 1 |  | 0 | 0 |  |  |
| 213 | SMM | LMS | 2 | 1 |  | 0 | 0 |  |  |
| 214 | SMM | LMS | 1 | 2 |  | 0 | 0 |  |  |
| 215 | SMM | LMS | 1 | 2 |  | 0 | 0 |  |  |
| 216 | SMM | LMS | 1 | 2 |  | 0 | 0 |  |  |
| 217 | SMM | LMS | 1 | 2 |  | 0 | 0 |  |  |
| 218 | SMM | LMS | 1 | 2 |  | 0 | 0 |  |  |
| 219 | SMM | LMS | 2 | 2 |  | 0 | 0 |  |  |
| 220 | SMM | LMS | 2 | 2 |  | 0 | 0 |  |  |
| 221 | SMM | LMS | 2 | 2 |  | 0 | 0 |  |  |
| 222 | SMM | LMS | 2 | 2 |  | 0 | 0 |  |  |
| 223 | SMM | LMS | 2 | 2 |  | 0 | 0 |  |  |
| 224 | SMM | LMS | 1 | 3 |  | 0 | 0 |  |  |
| 225 | SMM | LMS | 2 | 3 |  | 0 | 0 |  |  |
| 226 | SMM | LMS | 0 | 0 |  | 0 | 0 |  |  |
| 227 | SMM | LMS | 0 | 0 |  | 0 | 0 |  |  |
| 228 | SMM | LMS | 0 | 0 |  | 0 | 0 |  |  |
| 229 | SMM | LMS | 0 | 0 |  | 0 | 0 |  |  |
| 230 | SMM | LMS | 0 | 0 |  | 0 | 0 |  |  |
| 231 | SMM | LMS | 0 | 0 |  | 0 | 0 |  |  |
| 232 | SMM | LMS | 0 | 0 |  | 0 | 0 |  |  |
| 233 | SMM | LMS | 0 | 0 |  | 0 | 0 |  |  |
| 234 | SMM | LMS | 0 | 0 |  | 0 | 0 |  |  |
| 235 | SMM | LMS | 0 | 0 |  | 0 | 0 |  |  |
| 236 | SMM | LMS | 0 | 0 |  | 0 | 0 |  |  |
| 237 | SMM | LMS | 0 | 0 |  | 0 | 0 |  |  |
| 238 | SMM | LMS | 0 | 0 |  | 0 | 0 |  |  |
| 239 | SMM | LMS | 0 | 0 |  | 0 | 0 |  |  |
| 240 | SMM | LMS | 0 | 0 |  | 0 | 0 |  |  |
| 241 | SMM | LMS | 0 | 0 |  | 0 | 0 |  |  |
| 242 | SMM | LMS | 0 | 0 |  | 0 | 0 |  |  |
| 243 | SMM | LMS | 0 | 0 |  | 0 | 0 |  |  |
| 244 | SMM | LMS | 0 | 0 |  | 0 | 0 |  |  |
| 245 | SMM | LMS | 0 | 0 |  | 0 | 0 |  |  |
| 246 | SMM | LMS | 0 | 0 |  | 0 | 0 |  |  |
| 247 | SMM | LMS | 0 | 0 |  | 0 | 0 |  |  |
| 248 | SMM | LMS | 0 | 0 |  | 0 | 0 |  |  |
| 249 | SMM | LMS | 0 | 0 |  | 0 | 0 |  |  |
| 250 | SMM | LMS | 0 | 0 |  | 0 | 0 |  |  |
| 251 | SMM | LMS | 0 | 0 |  | 0 | 0 |  |  |
| 252 | SMM | LMS | 0 | 0 |  | 0 | 0 |  |  |
| 253 | SMM | LMS | 0 | 0 |  | 0 | 0 |  |  |
| 254 | SMM | LMS | 0 | 0 |  | 0 | 0 |  |  |
| 255 | SMM | LMS | 0 | 0 |  | 0 | 0 |  |  |
| 256 | SMM | LMS | 0 | 0 |  | 0 | 0 |  |  |
| 257 | SMM | LMS | 0 | 0 |  | 0 | 0 |  |  |
| 258 | SMM | LMS | 0 | 0 |  | 0 | 0 |  |  |
| 259 | SMM | LMS | 0 | 0 |  | 0 | 0 |  |  |
| 260 | SMM | LMS | 0 | 0 |  | 0 | 0 |  |  |
| 261 | SMM | LMS | 0 | 0 |  | 0 | 0 |  |  |
| 262 | SMM | LMS | 0 | 0 |  | 0 | 0 |  |  |
| 263 | SMM | LMS | 0 | 0 |  | 0 | 0 |  |  |
| 264 | SMM | LMS | 0 | 0 |  | 0 | 0 |  |  |
| 265 | SMM | LMS | 0 | 0 |  | 0 | 0 |  |  |
| 266 | SMM | LMS | 0 | 0 |  | 0 | 0 |  |  |
| 267 | SMM | LMS | 0 | 0 |  | 0 | 0 |  |  |
| 268 | SMM | LMS | 0 | 0 |  | 0 | 0 |  |  |
| 269 | SMM | LMS | 0 | 0 |  | 0 | 0 |  |  |
| 270 | SMM | LMS | 0 | 0 |  | 0 | 0 |  |  |
| 271 | SMM | LM | 0 | 0 |  | 0 | 0 |  |  |
| 272 | SMM | LM | 0 | 0 |  | 0 | 0 |  |  |
| 273 | SMM | LM | 0 | 0 |  | 0 | 0 |  |  |
| 274 | SMM | LM | 0 | 0 |  | 0 | 0 |  |  |
| 275 | SMM | LM | 0 | 0 |  | 0 | 0 |  |  |
| 276 | SMM | LM | 0 | 0 |  | 0 | 0 |  |  |
| 277 | Vascular | ASA | 0 | 0 |  | 0 | 0 |  |  |
| 278 | Vascular | ASA | 2 | 2 |  | 0 | 0 |  |  |
| 279 | Vascular | ASA | 2 | 2 |  | 0 | 0 |  |  |
| 280 | Vascular | ASA | 2 | 3 |  | 0 | 0 |  |  |
| 281 | Vascular | ASA | 0 | 0 |  | 0 | 0 |  |  |
| 282 | Vascular | ASA | 0 | 0 |  | 0 | 0 |  |  |
| 283 | Vascular | ASA | 0 | 0 |  | 0 | 0 |  |  |
| 284 | Vascular | ASA | 0 | 0 |  | 0 | 0 |  |  |
| 285 | Vascular | ASA | 0 | 0 |  | 0 | 0 |  |  |
| 286 | Vascular | ASA | 0 | 0 |  | 0 | 0 |  |  |
| 287 | Vascular | ASA | 0 | 0 |  | 0 | 0 |  |  |
| 288 | Vascular | ASA | 0 | 0 |  | 0 | 0 |  |  |
| 289 | Vascular | ASA | 0 | 0 |  | 0 | 0 |  |  |
| 290 | Vascular | ASA | 0 | 0 |  | 0 | 0 |  |  |
| 291 | Vascular | ASA | 0 | 0 |  | 0 | 0 |  |  |
| 292 | Vascular | ASA | 0 | 0 |  | 0 | 0 |  |  |
| 293 | Vascular | ASA | 0 | 0 |  | 0 | 0 |  |  |
| 294 | Vascular | ASA | 0 | 0 |  | 0 | 0 |  |  |
| 295 | Vascular | ASA | 0 | 0 |  | 0 | 0 |  |  |
| 296 | Vascular | ASA | 0 | 0 |  | 0 | 0 |  |  |
| 297 | Vascular | ASA | 0 | 0 |  | 0 | 0 |  |  |
| 298 | Vascular | ASA | 0 | 0 |  | 0 | 0 |  |  |
| 299 | Vascular | ASA | 0 | 0 |  | 0 | 0 |  |  |
| 300 | Vascular | ASA | 0 | 0 |  | 0 | 0 |  |  |
| 301 | Vascular | ASA | 0 | 0 |  | 0 | 0 |  |  |
| 302 | Vascular | ASA | 0 | 0 |  | 0 | 0 |  |  |
| 303 | Vascular | ASA | 0 | 0 |  | 0 | 0 |  |  |
| 304 | Vascular | ASA | 0 | 0 |  | 0 | 0 |  |  |
| 305 | Vascular | ASA | 0 | 0 |  | 0 | 0 |  |  |
| 306 | Vascular | HM | 1 | 1 |  | 0 | 0 |  |  |
| 307 | Vascular | HM | 1 | 1 |  | 0 | 0 |  |  |
| 308 | Vascular | HM | 0 | 0 |  | 0 | 0 |  |  |
| 309 | Vascular | HM | 0 | 0 |  | 0 | 0 |  |  |
| 310 | Vascular | HM | 0 | 0 |  | 0 | 0 |  |  |
| 311 | Vascular | HM | 0 | 0 |  | 0 | 0 |  |  |
| 312 | NS | MPNST | 1 | 1 |  | 0 | 0 |  |  |
| 313 | NS | MPNST | 1 | 2 |  | 0 | 0 |  |  |
| 314 | NS | MPNST | 1 | 2 |  | 0 | 0 |  |  |
| 315 | NS | MPNST | 2 | 2 |  | 0 | 0 |  |  |
| 316 | NS | MPNST | 1 | 3 |  | 0 | 0 |  |  |
| 317 | NS | MPNST | 2 | 3 |  | 0 | 0 |  |  |
| 318 | NS | MPNST | 2 | 3 |  | 0 | 0 |  |  |
| 319 | NS | MPNST | 0 | 0 |  | 0 | 0 |  |  |
| 320 | NS | MPNST | 0 | 0 |  | 0 | 0 |  |  |
| 321 | NS | MPNST | 0 | 0 |  | 0 | 0 |  |  |
| 322 | NS | MPNST | 0 | 0 |  | 0 | 0 |  |  |
| 323 | NS | MPNST | 0 | 0 |  | 0 | 0 |  |  |
| 324 | NS | MPNST | 0 | 0 |  | 0 | 0 |  |  |
| 325 | NS | MPNST | 0 | 0 |  | 0 | 0 |  |  |
| 326 | NS | MPNST | 0 | 0 |  | 0 | 0 |  |  |
| 327 | NS | MPNST | 0 | 0 |  | 0 | 0 |  |  |
| 328 | NS | MPNST | 0 | 0 |  | 0 | 0 |  |  |
| 329 | NS | MPNST | 0 | 0 |  | 0 | 0 |  |  |
| 330 | NS | MPNST | 0 | 0 |  | 0 | 0 |  |  |
| 331 | NS | MPNST | 0 | 0 |  | 0 | 0 |  |  |
| 332 | NS | MPNST | 0 | 0 |  | 0 | 0 |  |  |
| 333 | NS | SCHW | 1 | 1 |  | 0 | 0 |  |  |
| 334 | NS | SCHW | 1 | 1 |  | 0 | 0 |  |  |
| 335 | NS | SCHW | 2 | 2 |  | 0 | 0 |  |  |
| 336 | NS | SCHW | 0 | 0 |  | 0 | 0 |  |  |
| 337 | NS | SCHW | 0 | 0 |  | 0 | 0 |  |  |
| 338 | NS | SCHW | 0 | 0 |  | 0 | 0 |  |  |
| 339 | NS | SCHW | 0 | 0 |  | 0 | 0 |  |  |
| 340 | NS | SCHW | 0 | 0 |  | 0 | 0 |  |  |
| 341 | NS | SCHW | 0 | 0 |  | 0 | 0 |  |  |
| 342 | NS | SCHW | 0 | 0 |  | 0 | 0 |  |  |
| 343 | NS | SCHW | 0 | 0 |  | 0 | 0 |  |  |
| 344 | NS | SCHW | 0 | 0 |  | 0 | 0 |  |  |
| 345 | NS | SCHW | 0 | 0 |  | 0 | 0 |  |  |
| 346 | NS | SCHW | 0 | 0 |  | 0 | 0 |  |  |
| 347 | NS | NF | 0 | 0 |  | 0 | 0 |  |  |
| 348 | NS | NF | 0 | 0 |  | 0 | 0 |  |  |
| 349 | NS | GN | 0 | 0 |  | 0 | 0 |  |  |
| 350 | NS | GN | 0 | 0 |  | 0 | 0 |  |  |
| 351 | MFB | MFS | 1 | 2 |  | 0 | 0 |  |  |
| 352 | MFB | MFS | 0 | 0 |  | 0 | 0 |  |  |
| 353 | MFB | MFS | 0 | 0 |  | 0 | 0 |  |  |
| 354 | MFB | MFS | 0 | 0 |  | 0 | 0 |  |  |
| 355 | MFB | MFS | 0 | 0 |  | 0 | 0 |  |  |
| 356 | MFB | MFS | 0 | 0 |  | 0 | 0 |  |  |
| 357 | MFB | SFT | 1 | 1 |  | 0 | 0 |  |  |
| 358 | MFB | SFT | 1 | 1 |  | 0 | 0 |  |  |
| 359 | MFB | SFT | 1 | 1 |  | 0 | 0 |  |  |
| 360 | MFB | SFT | 1 | 1 |  | 0 | 0 |  |  |
| 361 | MFB | SFT | 1 | 1 |  | 0 | 0 |  |  |
| 362 | MFB | SFT | 1 | 1 |  | 0 | 0 |  |  |
| 363 | MFB | SFT | 1 | 1 |  | 0 | 0 |  |  |
| 364 | MFB | SFT | 1 | 2 |  | 0 | 0 |  |  |
| 365 | MFB | SFT | 1 | 2 |  | 0 | 0 |  |  |
| 366 | MFB | SFT | 1 | 2 |  | 0 | 0 |  |  |
| 367 | MFB | SFT | 1 | 2 |  | 0 | 0 |  |  |
| 368 | MFB | SFT | 1 | 3 |  | 0 | 0 |  |  |
| 369 | MFB | SFT | 0 | 0 |  | 0 | 0 |  |  |
| 370 | MFB | SFT | 0 | 0 |  | 0 | 0 |  |  |
| 371 | MFB | SFT | 0 | 0 |  | 0 | 0 |  |  |
| 372 | MFB | SFT | 0 | 0 |  | 0 | 0 |  |  |
| 373 | MFB | SFT | 0 | 0 |  | 0 | 0 |  |  |
| 374 | MFB | SFT | 0 | 0 |  | 0 | 0 |  |  |
| 375 | MFB | SFT | 0 | 0 |  | 0 | 0 |  |  |
| 376 | MFB | SFT | 0 | 0 |  | 0 | 0 |  |  |
| 377 | MFB | SFT | 0 | 0 |  | 0 | 0 |  |  |
| 378 | MFB | SFT | 0 | 0 |  | 0 | 0 |  |  |
| 379 | MFB | SFT | 0 | 0 |  | 0 | 0 |  |  |
| 380 | MFB | SFT | 0 | 0 |  | 0 | 0 |  |  |
| 381 | MFB | SFT | 0 | 0 |  | 0 | 0 |  |  |
| 382 | MFB | SFT | 0 | 0 |  | 0 | 0 |  |  |
| 383 | MFB | SFT | 0 | 0 |  | 0 | 0 |  |  |
| 384 | MFB | SFT | 0 | 0 |  | 0 | 0 |  |  |
| 385 | MFB | SFT | 0 | 0 |  | 0 | 0 |  |  |
| 386 | MFB | SFT | 0 | 0 |  | 0 | 0 |  |  |
| 387 | MFB | SFT | 0 | 0 |  | 0 | 0 |  |  |
| 388 | MFB | SFT | 0 | 0 |  | 0 | 0 |  |  |
| 389 | MFB | SFT | 0 | 0 |  | 0 | 0 |  |  |
| 390 | MFB | SFT | 0 | 0 |  | 0 | 0 |  |  |
| 391 | MFB | SFT | 0 | 0 |  | 0 | 0 |  |  |
| 392 | MFB | IMFT | 0 | 0 |  | 0 | 0 |  |  |
| 393 | MFB | IMFT | 0 | 0 |  | 0 | 0 |  |  |
| 394 | MFB | DES | 1 | 1 |  | 0 | 0 |  |  |
| 395 | MFB | DES | 1 | 1 |  | 0 | 0 |  |  |
| 396 | MFB | DES | 1 | 1 |  | 0 | 0 |  |  |
| 397 | MFB | DES | 1 | 1 |  | 0 | 0 |  |  |
| 398 | MFB | DES | 1 | 1 |  | 0 | 0 |  |  |
| 399 | MFB | DES | 1 | 2 |  | 0 | 0 |  |  |
| 400 | MFB | DES | 1 | 2 |  | 0 | 0 |  |  |
| 401 | MFB | DES | 1 | 2 |  | 0 | 0 |  |  |
| 402 | MFB | DES | 1 | 3 |  | 0 | 0 |  |  |
| 403 | MFB | DES | 1 | 3 |  | 0 | 0 |  |  |
| 404 | MFB | DES | 0 | 0 |  | 0 | 0 |  |  |
| 405 | MFB | DES | 0 | 0 |  | 0 | 0 |  |  |
| 406 | MFB | DES | 0 | 0 |  | 0 | 0 |  |  |
| 407 | MFB | DES | 0 | 0 |  | 0 | 0 |  |  |
| 408 | MFB | DES | 0 | 0 |  | 0 | 0 |  |  |
| 409 | MFB | DES | 0 | 0 |  | 0 | 0 |  |  |
| 410 | MFB | DES | 0 | 0 |  | 0 | 0 |  |  |
| 411 | MFB | DES | 0 | 0 |  | 0 | 0 |  |  |
| 412 | MFB | DES | 0 | 0 |  | 0 | 0 |  |  |
| 413 | MFB | DES | 0 | 0 |  | 0 | 0 |  |  |
| 414 | MFB | DES | 0 | 0 |  | 0 | 0 |  |  |
| 415 | MFB | DES | 0 | 0 |  | 0 | 0 |  |  |
| 416 | MFB | DES | 0 | 0 |  | 0 | 0 |  |  |
| 417 | MFB | DES | 0 | 0 |  | 0 | 0 |  |  |
| 418 | MFB | DES | 0 | 0 |  | 0 | 0 |  |  |
| 419 | MFB | DES | 0 | 0 |  | 0 | 0 |  |  |
| 420 | MFB | DES | 0 | 0 |  | 0 | 0 |  |  |
| 421 | MFB | DES | 0 | 0 |  | 0 | 0 |  |  |
| 422 | MFB | DES | 0 | 0 |  | 0 | 0 |  |  |
| 423 | MFB | DES | 0 | 0 |  | 0 | 0 |  |  |
| 424 | MFB | DES | 0 | 0 |  | 0 | 0 |  |  |
| 425 | MFB | DES | 0 | 0 |  | 0 | 0 |  |  |
| 426 | MFB | DES | 0 | 0 |  | 0 | 0 |  |  |
| 427 | MFB | DES | 0 | 0 |  | 0 | 0 |  |  |
| 428 | MFB | DES | 0 | 0 |  | 0 | 0 |  |  |
| 429 | MFB | DES | 0 | 0 |  | 0 | 0 |  |  |
| 430 | MFB | DES | 0 | 0 |  | 0 | 0 |  |  |
| 431 | MFB | DES | 0 | 0 |  | 0 | 0 |  |  |
| 432 | MFB | DES | 0 | 0 |  | 0 | 0 |  |  |
| 433 | MFB | DES | 0 | 0 |  | 0 | 0 |  |  |
| 434 | MFB | DES | 0 | 0 |  | 0 | 0 |  |  |
| 435 | MFB | DES | 0 | 0 |  | 0 | 0 |  |  |
| 436 | MFB | DES | 0 | 0 |  | 0 | 0 |  |  |
| 437 | MFB | DES | 0 | 0 |  | 0 | 0 |  |  |
| 438 | BT | ES | 1 | 1 |  | 0 | 0 |  |  |
| 439 | BT | ES | 1 | 1 |  | 0 | 0 |  |  |
| 440 | BT | ES | 1 | 1 |  | 0 | 0 |  |  |
| 441 | BT | ES | 1 | 1 |  | 0 | 0 |  |  |
| 442 | BT | ES | 1 | 1 |  | 0 | 0 |  |  |
| 443 | BT | ES | 2 | 1 |  | 0 | 0 |  |  |
| 444 | BT | ES | 2 | 1 |  | 0 | 0 |  |  |
| 445 | BT | ES | 1 | 2 |  | 0 | 0 |  |  |
| 446 | BT | ES | 2 | 2 |  | 0 | 0 |  |  |
| 447 | BT | ES | 2 | 2 |  | 0 | 0 |  |  |
| 448 | BT | ES | 2 | 3 |  | 0 | 0 |  |  |
| 449 | BT | ES | 2 | 3 |  | 0 | 0 |  |  |
| 450 | BT | ES | 2 | 3 |  | 0 | 0 |  |  |
| 451 | BT | ES | 2 | 3 |  | 0 | 0 |  |  |
| 452 | BT | ES | 0 | 0 |  | 0 | 0 |  |  |
| 453 | BT | ES | 0 | 0 |  | 0 | 0 |  |  |
| 454 | BT | ES | 0 | 0 |  | 0 | 0 |  |  |
| 455 | BT | ES | 0 | 0 |  | 0 | 0 |  |  |
| 456 | BT | ES | 0 | 0 |  | 0 | 0 |  |  |
| 457 | BT | ES | 0 | 0 |  | 0 | 0 |  |  |
| 458 | BT | ES | 0 | 0 |  | 0 | 0 |  |  |
| 459 | BT | ES | 0 | 0 |  | 0 | 0 |  |  |
| 460 | BT | ES | 0 | 0 |  | 0 | 0 |  |  |
| 461 | BT | ES | 0 | 0 |  | 0 | 0 |  |  |
| 462 | BT | ES | 0 | 0 |  | 0 | 0 |  |  |
| 463 | BT | ES | 0 | 0 |  | 0 | 0 |  |  |
| 464 | BT | ES | 0 | 0 |  | 0 | 0 |  |  |
| 465 | BT | ES | 0 | 0 |  | 0 | 0 |  |  |
| 466 | BT | ES | 0 | 0 |  | 0 | 0 |  |  |
| 467 | BT | ES | 0 | 0 |  | 0 | 0 |  |  |
| 468 | BT | ES | 0 | 0 |  | 0 | 0 |  |  |
| 469 | BT | ES | 0 | 0 |  | 0 | 0 |  |  |
| 470 | BT | ES | 0 | 0 |  | 0 | 0 |  |  |
| 471 | BT | ES | 0 | 0 |  | 0 | 0 |  |  |
| 472 | BT | ES | 0 | 0 |  | 0 | 0 |  |  |
| 473 | BT | ES | 0 | 0 |  | 0 | 0 |  |  |
| 474 | BT | ES | 0 | 0 |  | 0 | 0 |  |  |
| 475 | BT | ES | 0 | 0 |  | 0 | 0 |  |  |
| 476 | BT | ES | 0 | 0 |  | 0 | 0 |  |  |
| 477 | BT | ES | 0 | 0 |  | 0 | 0 |  |  |
| 478 | BT | ES | 0 | 0 |  | 0 | 0 |  |  |
| 479 | BT | ES | 0 | 0 |  | 0 | 0 |  |  |
| 480 | BT | ES | 0 | 0 |  | 0 | 0 |  |  |
| 481 | BT | ES | 0 | 0 |  | 0 | 0 |  |  |
| 482 | BT | ES | 0 | 0 |  | 0 | 0 |  |  |
| 483 | BT | ES | 0 | 0 |  | 0 | 0 |  |  |
| 484 | BT | ES | 0 | 0 |  | 0 | 0 |  |  |
| 485 | BT | ES | 0 | 0 |  | 0 | 0 |  |  |
| 486 | BT | ES | 0 | 0 |  | 0 | 0 |  |  |
| 487 | BT | ES | 0 | 0 |  | 0 | 0 |  |  |
| 488 | BT | ES | 0 | 0 |  | 0 | 0 |  |  |
| 489 | BT | ES | 0 | 0 |  | 0 | 0 |  |  |
| 490 | BT | ES | 0 | 0 |  | 0 | 0 |  |  |
| 491 | BT | ES | 0 | 0 |  | 0 | 0 |  |  |
| 492 | BT | ES | 0 | 0 |  | 0 | 0 |  |  |
| 493 | BT | ES | 0 | 0 |  | 0 | 0 |  |  |
| 494 | BT | ES | 0 | 0 |  | 0 | 0 |  |  |
| 495 | BT | ES | 0 | 0 |  | 0 | 0 |  |  |
| 496 | BT | ES | 0 | 0 |  | 0 | 0 |  |  |
| 497 | BT | ES | 0 | 0 |  | 0 | 0 |  |  |
| 498 | BT | ES | 0 | 0 |  | 0 | 0 |  |  |
| 499 | BT | ES | 0 | 0 |  | 0 | 0 |  |  |
| 500 | BT | ES | 0 | 0 |  | 0 | 0 |  |  |
| 501 | BT | ES | 0 | 0 |  | 0 | 0 |  |  |
| 502 | BT | ES | 0 | 0 |  | 0 | 0 |  |  |
| 503 | BT | ES | 0 | 0 |  | 0 | 0 |  |  |
| 504 | BT | ES | 0 | 0 |  | 0 | 0 |  |  |
| 505 | BT | ES | 0 | 0 |  | 0 | 0 |  |  |
| 506 | BT | ES | 0 | 0 |  | 0 | 0 |  |  |
| 507 | BT | ES | 0 | 0 |  | 0 | 0 |  |  |
| 508 | BT | ES | 0 | 0 |  | 0 | 0 |  |  |
| 509 | BT | ES | 0 | 0 |  | 0 | 0 |  |  |
| 510 | BT | ES | 0 | 0 |  | 0 | 0 |  |  |
| 511 | BT | ES | 0 | 0 |  | 0 | 0 |  |  |
| 512 | BT | ES | 0 | 0 |  | 0 | 0 |  |  |
| 513 | BT | ES | 0 | 0 |  | 0 | 0 |  |  |
| 514 | BT | ES | 0 | 0 |  | 0 | 0 |  |  |
| 515 | BT | ES | 0 | 0 |  | 0 | 0 |  |  |
| 516 | BT | ES | 0 | 0 |  | 0 | 0 |  |  |
| 517 | BT | ES | 0 | 0 |  | 0 | 0 |  |  |
| 518 | BT | ES | 0 | 0 |  | 0 | 0 |  |  |
| 519 | BT | ES | 0 | 0 |  | 0 | 0 |  |  |
| 520 | BT | ES | 0 | 0 |  | 0 | 0 |  |  |
| 521 | BT | ES | 0 | 0 |  | 0 | 0 |  |  |
| 522 | BT | ES | 0 | 0 |  | 0 | 0 |  |  |
| 523 | BT | ES | 0 | 0 |  | 0 | 0 |  |  |
| 524 | BT | ES | 0 | 0 |  | 0 | 0 |  |  |
| 525 | BT | ES | 0 | 0 |  | 0 | 0 |  |  |
| 526 | BT | ES | 0 | 0 |  | 0 | 0 |  |  |
| 527 | BT | ES | 0 | 0 |  | 0 | 0 |  |  |
| 528 | BT | ES | 0 | 0 |  | 0 | 0 |  |  |
| 529 | BT | ES | 0 | 0 |  | 0 | 0 |  |  |
| 530 | BT | ES | 0 | 0 |  | 0 | 0 |  |  |
| 531 | BT | ES | 0 | 0 |  | 0 | 0 |  |  |
| 532 | BT | ES | 0 | 0 |  | 0 | 0 |  |  |
| 533 | BT | ES | 0 | 0 |  | 0 | 0 |  |  |
| 534 | BT | ES | 0 | 0 |  | 0 | 0 |  |  |
| 535 | BT | ES | 0 | 0 |  | 0 | 0 |  |  |
| 536 | BT | ES | 0 | 0 |  | 0 | 0 |  |  |
| 537 | BT | ES | 0 | 0 |  | 0 | 0 |  |  |
| 538 | BT | ES | 0 | 0 |  | 0 | 0 |  |  |
| 539 | BT | ES | 0 | 0 |  | 0 | 0 |  |  |
| 540 | BT | ES | 0 | 0 |  | 0 | 0 |  |  |
| 541 | BT | ES | 0 | 0 |  | 0 | 0 |  |  |
| 542 | BT | ES | 0 | 0 |  | 0 | 0 |  |  |
| 543 | BT | ES | 0 | 0 |  | 0 | 0 |  |  |
| 544 | GIST | GIST | 1 | 1 |  | 0 | 0 |  |  |
| 545 | GIST | GIST | 1 | 1 |  | 0 | 0 |  |  |
| 546 | GIST | GIST | 1 | 1 |  | 0 | 0 |  |  |
| 547 | GIST | GIST | 1 | 1 |  | 0 | 0 |  |  |
| 548 | GIST | GIST | 1 | 1 |  | 0 | 0 |  |  |
| 549 | GIST | GIST | 1 | 1 |  | 0 | 0 |  |  |
| 550 | GIST | GIST | 1 | 1 |  | 0 | 0 |  |  |
| 551 | GIST | GIST | 1 | 1 |  | 0 | 0 |  |  |
| 552 | GIST | GIST | 1 | 1 |  | 0 | 0 |  |  |
| 553 | GIST | GIST | 1 | 1 |  | 0 | 0 |  |  |
| 554 | GIST | GIST | 1 | 1 |  | 0 | 0 |  |  |
| 555 | GIST | GIST | 1 | 1 |  | 0 | 0 |  |  |
| 556 | GIST | GIST | 2 | 1 |  | 0 | 0 |  |  |
| 557 | GIST | GIST | 1 | 2 |  | 0 | 0 |  |  |
| 558 | GIST | GIST | 1 | 2 |  | 0 | 0 |  |  |
| 559 | GIST | GIST | 1 | 2 |  | 0 | 0 |  |  |
| 560 | GIST | GIST | 1 | 2 |  | 0 | 0 |  |  |
| 561 | GIST | GIST | 1 | 2 |  | 0 | 0 |  |  |
| 562 | GIST | GIST | 1 | 2 |  | 0 | 0 |  |  |
| 563 | GIST | GIST | 1 | 2 |  | 0 | 0 |  |  |
| 564 | GIST | GIST | 1 | 2 |  | 0 | 0 |  |  |
| 565 | GIST | GIST | 2 | 2 |  | 0 | 0 |  |  |
| 566 | GIST | GIST | 2 | 3 |  | 0 | 0 |  |  |
| 567 | GIST | GIST | 0 | 0 |  | 0 | 0 |  |  |
| 568 | GIST | GIST | 0 | 0 |  | 0 | 0 |  |  |
| 569 | GIST | GIST | 0 | 0 |  | 0 | 0 |  |  |
| 570 | GIST | GIST | 0 | 0 |  | 0 | 0 |  |  |
| 571 | GIST | GIST | 0 | 0 |  | 0 | 0 |  |  |
| 572 | GIST | GIST | 0 | 0 |  | 0 | 0 |  |  |
| 573 | GIST | GIST | 0 | 0 |  | 0 | 0 |  |  |
| 574 | GIST | GIST | 0 | 0 |  | 0 | 0 |  |  |
| 575 | GIST | GIST | 0 | 0 |  | 0 | 0 |  |  |
| 576 | GIST | GIST | 0 | 0 |  | 0 | 0 |  |  |
| 577 | GIST | GIST | 0 | 0 |  | 0 | 0 |  |  |
| 578 | GIST | GIST | 0 | 0 |  | 0 | 0 |  |  |
| 579 | GIST | GIST | 0 | 0 |  | 0 | 0 |  |  |
| 580 | GIST | GIST | 0 | 0 |  | 0 | 0 |  |  |
| 581 | GIST | GIST | 0 | 0 |  | 0 | 0 |  |  |
| 582 | GIST | GIST | 0 | 0 |  | 0 | 0 |  |  |
| 583 | GIST | GIST | 0 | 0 |  | 0 | 0 |  |  |
| 584 | GIST | GIST | 0 | 0 |  | 0 | 0 |  |  |
| 585 | GIST | GIST | 0 | 0 |  | 0 | 0 |  |  |
| 586 | GIST | GIST | 0 | 0 |  | 0 | 0 |  |  |
| 587 | GIST | GIST | 0 | 0 |  | 0 | 0 |  |  |
| 588 | GIST | GIST | 0 | 0 |  | 0 | 0 |  |  |
| 589 | GIST | GIST | 0 | 0 |  | 0 | 0 |  |  |
| 590 | GIST | GIST | 0 | 0 |  | 0 | 0 |  |  |
| 591 | GIST | GIST | 0 | 0 |  | 0 | 0 |  |  |
| 592 | GIST | GIST | 0 | 0 |  | 0 | 0 |  |  |
| 593 | GIST | GIST | 0 | 0 |  | 0 | 0 |  |  |
| 594 | GIST | GIST | 0 | 0 |  | 0 | 0 |  |  |
| 595 | GIST | GIST | 0 | 0 |  | 0 | 0 |  |  |
| 596 | GIST | GIST | 0 | 0 |  | 0 | 0 |  |  |
| 597 | GIST | GIST | 0 | 0 |  | 0 | 0 |  |  |
| 598 | GIST | GIST | 0 | 0 |  | 0 | 0 |  |  |
| 599 | GIST | GIST | 0 | 0 |  | 0 | 0 |  |  |
| 600 | GIST | GIST | 0 | 0 |  | 0 | 0 |  |  |
| 601 | GIST | GIST | 0 | 0 |  | 0 | 0 |  |  |
| 602 | GIST | GIST | 0 | 0 |  | 0 | 0 |  |  |
| 603 | GIST | GIST | 0 | 0 |  | 0 | 0 |  |  |
| 604 | GIST | GIST | 0 | 0 |  | 0 | 0 |  |  |
| 605 | GIST | GIST | 0 | 0 |  | 0 | 0 |  |  |
| 606 | GIST | GIST | 0 | 0 |  | 0 | 0 |  |  |
| 607 | GIST | GIST | 0 | 0 |  | 0 | 0 |  |  |
| 608 | GIST | GIST | 0 | 0 |  | 0 | 0 |  |  |
| 609 | GIST | GIST | 0 | 0 |  | 0 | 0 |  |  |
| 610 | GIST | GIST | 0 | 0 |  | 0 | 0 |  |  |
| 611 | GIST | GIST | 0 | 0 |  | 0 | 0 |  |  |
| 612 | GIST | GIST | 0 | 0 |  | 0 | 0 |  |  |
| 613 | GIST | GIST | 0 | 0 |  | 0 | 0 |  |  |
| 614 | GIST | GIST | 0 | 0 |  | 0 | 0 |  |  |
| 615 | GIST | GIST | 0 | 0 |  | 0 | 0 |  |  |
| 616 | GIST | GIST | 0 | 0 |  | 0 | 0 |  |  |
| 617 | GIST | GIST | 0 | 0 |  | 0 | 0 |  |  |
| 618 | GIST | GIST | 0 | 0 |  | 0 | 0 |  |  |
| 619 | GIST | GIST | 0 | 0 |  | 0 | 0 |  |  |
| 620 | GIST | GIST | 0 | 0 |  | 0 | 0 |  |  |
| 621 | GIST | GIST | 0 | 0 |  | 0 | 0 |  |  |
| 622 | GIST | GIST | 0 | 0 |  | 0 | 0 |  |  |
| 623 | GIST | GIST | 0 | 0 |  | 0 | 0 |  |  |
| 624 | GIST | GIST | 0 | 0 |  | 0 | 0 |  |  |
| 625 | GIST | GIST | 0 | 0 |  | 0 | 0 |  |  |
| 626 | GIST | GIST | 0 | 0 |  | 0 | 0 |  |  |
| 627 | GIST | GIST | 0 | 0 |  | 0 | 0 |  |  |
| 628 | GIST | GIST | 0 | 0 |  | 0 | 0 |  |  |
| 629 | GIST | GIST | 0 | 0 |  | 0 | 0 |  |  |
| 630 | GIST | GIST | 0 | 0 |  | 0 | 0 |  |  |
| 631 | GIST | GIST | 0 | 0 |  | 0 | 0 |  |  |
| 632 | GIST | GIST | 0 | 0 |  | 0 | 0 |  |  |
| 633 | GIST | GIST | 0 | 0 |  | 0 | 0 |  |  |
| 634 | GIST | GIST | 0 | 0 |  | 0 | 0 |  |  |
| 635 | GIST | GIST | 0 | 0 |  | 0 | 0 |  |  |
| 636 | GIST | GIST | 0 | 0 |  | 0 | 0 |  |  |
| 637 | GIST | GIST | 0 | 0 |  | 0 | 0 |  |  |
| 638 | GIST | GIST | 0 | 0 |  | 0 | 0 |  |  |
| 639 | GIST | GIST | 0 | 0 |  | 0 | 0 |  |  |
| 640 | GIST | GIST | 0 | 0 |  | 0 | 0 |  |  |
| 641 | GIST | GIST | 0 | 0 |  | 0 | 0 |  |  |
| 642 | GIST | GIST | 0 | 0 |  | 0 | 0 |  |  |
| 643 | GIST | GIST | 0 | 0 |  | 0 | 0 |  |  |
| 644 | GIST | GIST | 0 | 0 |  | 0 | 0 |  |  |
| 645 | GIST | GIST | 0 | 0 |  | 0 | 0 |  |  |
| 646 | GIST | GIST | 0 | 0 |  | 0 | 0 |  |  |
| 647 | GIST | GIST | 0 | 0 |  | 0 | 0 |  |  |
| 648 | GIST | GIST | 0 | 0 |  | 0 | 0 |  |  |
| 649 | GIST | GIST | 0 | 0 |  | 0 | 0 |  |  |
| 650 | GIST | GIST | 0 | 0 |  | 0 | 0 |  |  |
| 651 | GIST | GIST | 0 | 0 |  | 0 | 0 |  |  |
| 652 | GIST | GIST | 0 | 0 |  | 0 | 0 |  |  |
| 653 | GIST | GIST | 0 | 0 |  | 0 | 0 |  |  |
| 654 | GIST | GIST | 0 | 0 |  | 0 | 0 |  |  |
| 655 | GIST | GIST | 0 | 0 |  | 0 | 0 |  |  |
| 656 | GIST | GIST | 0 | 0 |  | 0 | 0 |  |  |
| 657 | GIST | GIST | 0 | 0 |  | 0 | 0 |  |  |
| 658 | GIST | GIST | 0 | 0 |  | 0 | 0 |  |  |
| 659 | GIST | GIST | 0 | 0 |  | 0 | 0 |  |  |
| 660 | GIST | GIST | 0 | 0 |  | 0 | 0 |  |  |
| 661 | GIST | GIST | 0 | 0 |  | 0 | 0 |  |  |
| 662 | GIST | GIST | 0 | 0 |  | 0 | 0 |  |  |
| 623 | GIST | GIST | 0 | 0 |  | 0 | 0 |  |  |
| 624 | GIST | GIST | 0 | 0 |  | 0 | 0 |  |  |
| 625 | GIST | GIST | 0 | 0 |  | 0 | 0 |  |  |
| 626 | GIST | GIST | 0 | 0 |  | 0 | 0 |  |  |
| 627 | GIST | GIST | 0 | 0 |  | 0 | 0 |  |  |
| 628 | GIST | GIST | 0 | 0 |  | 0 | 0 |  |  |
| 629 | GIST | GIST | 0 | 0 |  | 0 | 0 |  |  |
| 630 | GIST | GIST | 0 | 0 |  | 0 | 0 |  |  |
| 631 | GIST | GIST | 0 | 0 |  | 0 | 0 |  |  |
| 632 | GIST | GIST | 0 | 0 |  | 0 | 0 |  |  |
| 633 | GIST | GIST | 0 | 0 |  | 0 | 0 |  |  |
| 634 | GIST | GIST | 0 | 0 |  | 0 | 0 |  |  |
| 635 | GIST | GIST | 0 | 0 |  | 0 | 0 |  |  |
| 636 | GIST | GIST | 0 | 0 |  | 0 | 0 |  |  |
| 637 | GIST | GIST | 0 | 0 |  | 0 | 0 |  |  |
| 638 | GIST | GIST | 0 | 0 |  | 0 | 0 |  |  |
| 639 | GIST | GIST | 0 | 0 |  | 0 | 0 |  |  |
| 640 | GIST | GIST | 0 | 0 |  | 0 | 0 |  |  |
| 641 | GIST | GIST | 0 | 0 |  | 0 | 0 |  |  |
| 642 | GIST | GIST | 0 | 0 |  | 0 | 0 |  |  |
| 643 | GIST | GIST | 0 | 0 |  | 0 | 0 |  |  |
| 644 | GIST | GIST | 0 | 0 |  | 0 | 0 |  |  |
| 645 | GIST | GIST | 0 | 0 |  | 0 | 0 |  |  |
| 646 | GIST | GIST | 0 | 0 |  | 0 | 0 |  |  |
| 647 | GIST | GIST | 0 | 0 |  | 0 | 0 |  |  |
| 648 | GIST | GIST | 0 | 0 |  | 0 | 0 |  |  |
| 649 | GIST | GIST | 0 | 0 |  | 0 | 0 |  |  |
| 650 | GIST | GIST | 0 | 0 |  | 0 | 0 |  |  |
| 651 | GIST | GIST | 0 | 0 |  | 0 | 0 |  |  |
| 652 | GIST | GIST | 0 | 0 |  | 0 | 0 |  |  |
| 653 | GIST | GIST | 0 | 0 |  | 0 | 0 |  |  |
| 654 | GIST | GIST | 0 | 0 |  | 0 | 0 |  |  |
| 655 | GIST | GIST | 0 | 0 |  | 0 | 0 |  |  |
| 656 | GIST | GIST | 0 | 0 |  | 0 | 0 |  |  |
| 657 | GIST | GIST | 0 | 0 |  | 0 | 0 |  |  |
| 658 | GIST | GIST | 0 | 0 |  | 0 | 0 |  |  |
| 659 | GIST | GIST | 0 | 0 |  | 0 | 0 |  |  |
| 660 | GIST | GIST | 0 | 0 |  | 0 | 0 |  |  |
| 661 | GIST | GIST | 0 | 0 |  | 0 | 0 |  |  |
| 662 | GIST | GIST | 0 | 0 |  | 0 | 0 |  |  |
| 663 | GIST | GIST | 0 | 0 |  | 0 | 0 |  |  |
| 664 | GIST | GIST | 0 | 0 |  | 0 | 0 |  |  |
| 665 | GIST | GIST | 0 | 0 |  | 0 | 0 |  |  |
| 666 | GIST | GIST | 0 | 0 |  | 0 | 0 |  |  |
| 667 | GIST | GIST | 0 | 0 |  | 0 | 0 |  |  |
| 668 | GIST | GIST | 0 | 0 |  | 0 | 0 |  |  |
| 669 | GIST | GIST | 0 | 0 |  | 0 | 0 |  |  |
| 670 | GIST | GIST | 0 | 0 |  | 0 | 0 |  |  |
| 671 | GIST | GIST | 0 | 0 |  | 0 | 0 |  |  |
| 672 | GIST | GIST | 0 | 0 |  | 0 | 0 |  |  |
| 673 | GIST | GIST | 0 | 0 |  | 0 | 0 |  |  |
| 674 | GIST | GIST | 0 | 0 |  | 0 | 0 |  |  |
| 675 | GIST | GIST | 0 | 0 |  | 0 | 0 |  |  |
| 676 | GIST | GIST | 0 | 0 |  | 0 | 0 |  |  |
| 677 | GIST | GIST | 0 | 0 |  | 0 | 0 |  |  |
| 678 | GIST | GIST | 0 | 0 |  | 0 | 0 |  |  |
| 679 | GIST | GIST | 0 | 0 |  | 0 | 0 |  |  |
| 680 | GIST | GIST | 0 | 0 |  | 0 | 0 |  |  |
| 681 | GIST | GIST | 0 | 0 |  | 0 | 0 |  |  |
| 682 | GIST | GIST | 0 | 0 |  | 0 | 0 |  |  |
| 683 | GIST | GIST | 0 | 0 |  | 0 | 0 |  |  |
| 684 | GIST | GIST | 0 | 0 |  | 0 | 0 |  |  |
| 685 | GIST | GIST | 0 | 0 |  | 0 | 0 |  |  |
| 686 | GIST | GIST | 0 | 0 |  | 0 | 0 |  |  |
| 687 | GIST | GIST | 0 | 0 |  | 0 | 0 |  |  |
| 688 | GIST | GIST | 0 | 0 |  | 0 | 0 |  |  |
| 689 | GIST | GIST | 0 | 0 |  | 0 | 0 |  |  |
| 690 | GIST | GIST | 0 | 0 |  | 0 | 0 |  |  |
| 691 | GIST | GIST | 0 | 0 |  | 0 | 0 |  |  |
| 692 | GIST | GIST | 0 | 0 |  | 0 | 0 |  |  |
| 693 | GIST | GIST | 0 | 0 |  | 0 | 0 |  |  |
| 694 | GIST | GIST | 0 | 0 |  | 0 | 0 |  |  |
| 695 | GIST | GIST | 0 | 0 |  | 0 | 0 |  |  |
| 696 | GIST | GIST | 0 | 0 |  | 0 | 0 |  |  |
| 697 | GIST | GIST | 0 | 0 |  | 0 | 0 |  |  |
| 698 | GIST | GIST | 0 | 0 |  | 0 | 0 |  |  |
| 699 | GIST | GIST | 0 | 0 |  | 0 | 0 |  |  |
| 700 | GIST | GIST | 0 | 0 |  | 0 | 0 |  |  |
| 701 | GIST | GIST | 0 | 0 |  | 0 | 0 |  |  |
| 702 | GIST | GIST | 0 | 0 |  | 0 | 0 |  |  |
| 703 | GIST | GIST | 0 | 0 |  | 0 | 0 |  |  |
| 704 | GIST | GIST | 0 | 0 |  | 0 | 0 |  |  |
| 705 | GIST | GIST | 0 | 0 |  | 0 | 0 |  |  |
| 706 | GIST | GIST | 0 | 0 |  | 0 | 0 |  |  |
| 707 | GIST | GIST | 0 | 0 |  | 0 | 0 |  |  |
| 708 | GIST | GIST | 0 | 0 |  | 0 | 0 |  |  |
| 709 | GIST | GIST | 0 | 0 |  | 0 | 0 |  |  |
| 710 | GIST | GIST | 0 | 0 |  | 0 | 0 |  |  |
| 711 | GIST | GIST | 0 | 0 |  | 0 | 0 |  |  |
| 712 | GIST | GIST | 0 | 0 |  | 0 | 0 |  |  |
| 713 | GIST | GIST | 0 | 0 |  | 0 | 0 |  |  |
| 714 | GIST | GIST | 0 | 0 |  | 0 | 0 |  |  |
| 715 | GIST | GIST | 0 | 0 |  | 0 | 0 |  |  |
| 716 | GIST | GIST | 0 | 0 |  | 0 | 0 |  |  |
| 717 | GIST | GIST | 0 | 0 |  | 0 | 0 |  |  |
| 718 | GIST | GIST | 0 | 0 |  | 0 | 0 |  |  |
| 719 | GIST | GIST | 0 | 0 |  | 0 | 0 |  |  |
| 720 | GIST | GIST | 0 | 0 |  | 0 | 0 |  |  |
| 721 | GIST | GIST | 0 | 0 |  | 0 | 0 |  |  |
| 722 | GIST | GIST | 0 | 0 |  | 0 | 0 |  |  |
| 723 | GIST | GIST | 0 | 0 |  | 0 | 0 |  |  |
| 724 | GIST | GIST | 0 | 0 |  | 0 | 0 |  |  |
| 725 | GIST | GIST | 0 | 0 |  | 0 | 0 |  |  |
| 726 | GIST | GIST | 0 | 0 |  | 0 | 0 |  |  |
| 727 | TUD | SS | 1 | 1 |  | 0 | 0 |  |  |
| 728 | TUD | SS | 1 | 2 |  | 0 | 0 |  |  |
| 729 | TUD | SS | 1 | 2 |  | 0 | 0 |  |  |
| 730 | TUD | SS | 2 | 2 |  | 0 | 0 |  |  |
| 731 | TUD | SS | 1 | 3 |  | 0 | 0 |  |  |
| 732 | TUD | SS | 1 | 3 |  | 0 | 0 |  |  |
| 733 | TUD | SS | 1 | 3 |  | 0 | 0 |  |  |
| 734 | TUD | SS | 1 | 3 |  | 0 | 0 |  |  |
| 735 | TUD | SS | 2 | 3 |  | 0 | 0 |  |  |
| 736 | TUD | SS | 0 | 0 |  | 0 | 0 |  |  |
| 737 | TUD | SS | 0 | 0 |  | 0 | 0 |  |  |
| 738 | TUD | SS | 0 | 0 |  | 0 | 0 |  |  |
| 739 | TUD | SS | 0 | 0 |  | 0 | 0 |  |  |
| 740 | TUD | SS | 0 | 0 |  | 0 | 0 |  |  |
| 741 | TUD | SS | 0 | 0 |  | 0 | 0 |  |  |
| 742 | TUD | SS | 0 | 0 |  | 0 | 0 |  |  |
| 743 | US | ESS | 1 | 2 |  | 0 | 0 |  |  |
| 744 | US | ESS | 0 | 0 |  | 0 | 0 |  |  |
| 745 | US | ESS | 0 | 0 |  | 0 | 0 |  |  |
| 746 | US | ESS | 0 | 0 |  | 0 | 0 |  |  |
| 747 | US | UPS | 1 | 1 |  | 0 | 0 |  |  |
| 748 | US | UPS | 1 | 1 |  | 0 | 0 |  |  |
| 749 | US | UPS | 1 | 1 |  | 0 | 0 |  |  |
| 750 | US | UPS | 1 | 2 |  | 0 | 0 |  |  |
| 751 | US | UPS | 1 | 2 |  | 0 | 0 |  |  |
| 752 | US | UPS | 1 | 2 |  | 0 | 0 |  |  |
| 753 | US | UPS | 1 | 2 |  | 0 | 0 |  |  |
| 754 | US | UPS | 1 | 2 |  | 0 | 0 |  |  |
| 755 | US | UPS | 1 | 2 |  | 0 | 0 |  |  |
| 756 | US | UPS | 2 | 2 |  | 0 | 0 |  |  |
| 757 | US | UPS | 2 | 2 |  | 0 | 0 |  |  |
| 758 | US | UPS | 1 | 3 |  | 0 | 0 |  |  |
| 759 | US | UPS | 2 | 3 |  | 0 | 0 |  |  |
| 760 | US | UPS | 2 | 3 |  | 0 | 0 |  |  |
| 761 | US | UPS | 2 | 3 |  | 0 | 0 |  |  |
| 762 | US | UPS | 0 | 0 |  | 0 | 0 |  |  |
| 763 | US | UPS | 0 | 0 |  | 0 | 0 |  |  |
| 764 | US | UPS | 0 | 0 |  | 0 | 0 |  |  |
| 765 | US | UPS | 0 | 0 |  | 0 | 0 |  |  |
| 766 | US | UPS | 0 | 0 |  | 0 | 0 |  |  |
| 767 | US | UPS | 0 | 0 |  | 0 | 0 |  |  |
| 768 | US | UPS | 0 | 0 |  | 0 | 0 |  |  |
| 769 | US | UPS | 0 | 0 |  | 0 | 0 |  |  |
| 770 | US | UPS | 0 | 0 |  | 0 | 0 |  |  |
| 771 | US | UPS | 0 | 0 |  | 0 | 0 |  |  |
| 772 | US | UPS | 0 | 0 |  | 0 | 0 |  |  |
| 773 | US | UPS | 0 | 0 |  | 0 | 0 |  |  |
| 774 | US | UPS | 0 | 0 |  | 0 | 0 |  |  |
| 775 | US | UPS | 0 | 0 |  | 0 | 0 |  |  |
| 776 | US | UPS | 0 | 0 |  | 0 | 0 |  |  |
| 777 | US | UPS | 0 | 0 |  | 0 | 0 |  |  |
| 778 | US | UPS | 0 | 0 |  | 0 | 0 |  |  |
| 779 | US | UPS | 0 | 0 |  | 0 | 0 |  |  |

* 0= no staining; 1=<5% of neovasculature or tumor staining for PSMA, 2=>5%of neovasculature or tumor staining for PSMA

**Abbreviations:**

WHO subtype: SKM, skeletal-muscle tumor; SMM, smooth-muscle tumor; NS, nerve sheath tumor, MFB, fibroblastic/myofibroblastic tumors; BT, bone tumor; GIST, gastrointestinal stromal tumor; TUD, tumors of uncertain differentiation; US, undifferentiated sarcoma.

Tumor type: WDLS, well-differentiated liposarcoma; DDLS, dedifferentiated liposarcoma; PLS, pleomorphic liposarcoma; MLS, myxoid liposarcoma; ERMS, embryonal rhabdomyosarcoma; ARMS, alveolar rhabdomyosarcoma; PRMS, pleomorphic rhabdomyosarcoma; LMS, leiomyosarcoma; LM, leiomyoma; ASA, angiosarcomas of soft tissue; HM, haemangioma; MPNST, malignant peripheral nerve sheath tumor; SCHW, schwannoma; NF, neurofibroma; GN, ganglioneuroma; MFS, myxofibrosarcoma; SFT, solitary fibrous tumor; IMFT, inflammatory myofibroblastic tumor; DES, desmoid-type fibromatosis; ES, Ewing Sarcoma; GIST, gastrointestinal stromal tumor; SS, synovial sarcoma; ESS, endometrial stromal sarcoma; UPS, undifferentiated pleomorphic sarcoma.
