# Supplementary material for: Expression of PSMA in tumor neovasculature of high grade sarcomas including synovial sarcoma, rhabdomyosarcoma, undifferentiated sarcoma and MPNST
Source: Oncotarget. 2016 Dec 16;8(3):4268–76. doi: 10.18632/oncotarget.13994 (PMC5354830; doi:10.18632/oncotarget.13994)
Supplement: Supplementary file 2 [file oncotarget-08-4268-s002.docx]

**Supplementary Table S2.** Comparison of Semi-quantitative immunohistochemistry results using TMA corresponding whole slides.

|  | |  |  | **TMA**  **PSMA Immunohistochemistry** | | | | | | **Whole slides**  **PSMA Immunohistochemistry** | | | | | |  |  |  |  |
| --- | --- | --- | --- | --- | --- | --- | --- | --- | --- | --- | --- | --- | --- | --- | --- | --- | --- | --- | --- |
| **No.** | **WHO - classification** | | **Tumor type** | **Neovasculature proportion*** | **Neovasculature intwensity [0-3]** | **Tumor cells proportion*** | **Tumor cells intensity [0-3]** |  | **Neovasculature proportion*** | | **Neovasculature intensity [0-3]** | **Tumor cells proportion*** | **Tumor cells intensity [0-3]** |  |  |  |  |  |  |
| 1 | | SMM | LM | 0 | 0 | 0 | 0 |  | 0 | | 0 | 0 | 0 |  |  |  |  |  |  |
| 2 | | SMM | LM | 0 | 0 | 0 | 0 |  | 0 | | 0 | 0 | 0 |  |  |  |  |  |  |
| 3 | | SMM | LM | 0 | 0 | 0 | 0 |  | 0 | | 0 | 0 | 0 |  |  |  |  |  |  |
| 4 | | SMM | LM | 0 | 0 | 0 | 0 |  | 0 | | 0 | 0 | 0 |  |  |  |  |  |  |
| 5 | | SMM | LM | 0 | 0 | 0 | 0 |  | 0 | | 0 | 0 | 0 |  |  |  |  |  |  |
| 6 | | SMM | LM | 0 | 0 | 0 | 0 |  | 0 | | 0 | 0 | 0 |  |  |  |  |  |  |
| 7 | | Vascular | HM | 0 | 0 | 0 | 0 |  | 1 | | 2 | 0 | 0 |  |  |  |  |  |  |
| 8 | | Vascular | HM | 0 | 0 | 0 | 0 |  | 0 | | 0 | 0 | 0 |  |  |  |  |  |  |
| 9 | | Vascular | HM | 0 | 0 | 0 | 0 |  | 0 | | 0 | 0 | 0 |  |  |  |  |  |  |
| 10 | | Vascular | HM | 0 | 0 | 0 | 0 |  | 0 | | 0 | 0 | 0 |  |  |  |  |  |  |
| 11 | | Vascular | HM | 0 | 0 | 0 | 0 |  | 0 | | 0 | 0 | 0 |  |  |  |  |  |  |
| 12 | | Vascular | HM | 0 | 0 | 0 | 0 |  | 0 | | 0 | 0 | 0 |  |  |  |  |  |  |
| 13 | | MFB | DES | 1 | 3 | 0 | 0 |  | 1 | | 3 | 0 | 0 |  |  |  |  |  |  |
| 14 | | MFB | DES | 1 | 1 | 0 | 0 |  | 1 | | 1 | 0 | 0 |  |  |  |  |  |  |
| 15 | | MFB | DES | 1 | 2 | 0 | 0 |  | 1 | | 2 | 0 | 0 |  |  |  |  |  |  |
| 16 | | MFB | DES | 0 | 0 | 0 | 0 |  | 0 | | 0 | 0 | 0 |  |  |  |  |  |  |
| 17 | | MFB | DES | 0 | 0 | 0 | 0 |  | 0 | | 0 | 0 | 0 |  |  |  |  |  |  |
| 18 | | MFB | DES | 0 | 0 | 0 | 0 |  | 0 | | 0 | 0 | 0 |  |  |  |  |  |  |
| 19 | | MFB | DES | 0 | 0 | 0 | 0 |  | 0 | | 0 | 0 | 0 |  |  |  |  |  |  |
| 20 | | MFB | DES | 0 | 0 | 0 | 0 |  | 0 | | 0 | 0 | 0 |  |  |  |  |  |  |
| 21 | | MFB | DES | 0 | 0 | 0 | 0 |  | 0 | | 0 | 0 | 0 |  |  |  |  |  |  |
| 22 | | MFB | DES | 0 | 0 | 0 | 0 |  | 0 | | 0 | 0 | 0 |  |  |  |  |  |  |
| 23 | | MFB | DES | 0 | 0 | 0 | 0 |  | 0 | | 0 | 0 | 0 |  |  |  |  |  |  |
| 24 | | MFB | DES | 0 | 0 | 0 | 0 |  | 0 | | 0 | 0 | 0 |  |  |  |  |  |  |
| 25 | | MFB | DES | 0 | 0 | 0 | 0 |  | 0 | | 0 | 0 | 0 |  |  |  |  |  |  |
| 26 | | NS | MPNST | 2 | 3 | 0 | 0 |  | 2 | | 3 | 0 | 0 |  |  |  |  |  |  |
| 27 | | NS | MPNST | 0 | 0 | 0 | 0 |  | 1 | | 1 | 0 | 0 |  |  |  |  |  |  |
| 28 | | NS | MPNST | 0 | 0 | 0 | 0 |  | 0 | | 0 | 0 | 0 |  |  |  |  |  |  |
| 29 | | NS | MPNST | 0 | 0 | 0 | 0 |  | 0 | | 0 | 0 | 0 |  |  |  |  |  |  |
| 30 | | NS | MPNST | 0 | 0 | 0 | 0 |  | 0 | | 0 | 0 | 0 |  |  |  |  |  |  |
| 31 | | NS | MPNST | 0 | 0 | 0 | 0 |  | 0 | | 0 | 0 | 0 |  |  |  |  |  |  |
| 32 | | NS | MPNST | 0 | 0 | 0 | 0 |  | 0 | | 0 | 0 | 0 |  |  |  |  |  |  |
| 33 | | TUD | SS | 1 | 3 | 0 | 0 |  | 1 | | 3 | 0 | 0 |  |  |  |  |  |  |
| 34 | | TUD | SS | 1 | 3 | 0 | 0 |  | 1 | | 3 | 0 | 0 |  |  |  |  |  |  |
| 35 | | TUD | SS | 0 | 0 | 0 | 0 |  | 0 | | 0 | 0 | 0 |  |  |  |  |  |  |
| 36 | | TUD | SS | 0 | 0 | 0 | 0 |  | 0 | | 0 | 0 | 0 |  |  |  |  |  |  |
| 37 | | TUD | SS | 0 | 0 | 0 | 0 |  | 0 | | 0 | 0 | 0 |  |  |  |  |  |  |

* 0= no staining; 1=<5% of neovasculature or tumor staining for PSMA, 2=>5%of neovasculature or tumor staining for PSMA

**Abbreviations:**

WHO subtypes: SMM, smooth muscle tumor; MFB, fibroblastic/myofibroblastic tumor; NS, nerve sheath tumor; TUD, tumor of uncertain differentiation

Tumor type: LM, leiomyoma; HM, haemangioma; DES, desmoid-type fibromatosis; MPNST, malignant peripheral nerve sheath tumor; SS, synvoial sarcoma
